# A Gene‐Switch Platform Interfacing with Reactive Oxygen Species Enables Transcription Fine‐Tuning by Soluble and Volatile Pharmacologics and Food Additives

**DOI:** 10.1002/advs.202306333

**Published:** 2024-03-25

**Authors:** Jinbo Huang, Shuai Xue, Ana Palma Teixeira, Martin Fussenegger

**Affiliations:** ^1^ Department of Biosystems Science and Engineering ETH Zurich Klingelbergstrasse 48 Basel CH‐4056 Switzerland; ^2^ Present address: Key Laboratory of Growth Regulation and Translational Research of Zhejiang Province School of Life Sciences, Westlake University Hangzhou, Zhejiang China; ^3^ Faculty of Science University of Basel Klingelbergstrasse 48 Basel CH‐4056 Switzerland

**Keywords:** diabetes, gene expression, gene switches, synthetic biology

## Abstract

Synthetic biology aims to engineer transgene switches for precise therapeutic protein control in cell‐based gene therapies. However, off‐the‐shelf trigger‐inducible gene circuits are usually switched on by single or structurally similar molecules. This study presents a mammalian gene‐switch platform that controls therapeutic gene expression by a wide range of molecules generating low, non‐toxic levels of reactive oxygen species (ROS). In this system, KEAP1 (Kelch‐like ECH‐associated protein 1) serves as ROS sensor, regulating the translocation of NRF2 (nuclear factor erythroid 2‐related factor 2) to the nucleus, where NRF2 binds to  antioxidant response elements (ARE) to activate the expression of a gene of interest. It is found that a promoter containing eight‐tandem ARE repeats is highly sensitive to the low ROS levels generated by the soluble and volatile molecules, which include food preservatives, food additives, pharmaceuticals, and signal transduction inducers. In a proof‐of‐concept study, it is shown that many of these compounds can independently trigger microencapsulated engineered cells to produce sufficient insulin to restore normoglycemia in experimental type‐1 diabetic mice. It is believed that this system greatly extends the variety of small‐molecule inducers available to drive therapeutic gene switches.

## Graphical abstract

ROS‐enhancing molecules regulate therapeutic protein expression in mammalian cells. Co‐expression of KEAP1 and NRF2 forms a complex in low‐ROS conditions. Introduction of ROS‐enhancing molecules elevates ROS levels, dissociating the KEAP1/NRF2 complex. NRF2 translocates to the nucleus, activating transgene expression via antioxidant response element (ARE). Alginated‐encapsulated engineered cells implanted intraperitoneally into mice control the therapeutic protein production in vivo.

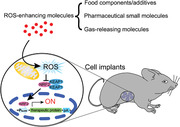

## 1 Introduction

Synthetic biology has dramatically advanced the rational design and engineering of trigger‐inducible gene switches that enable programming of novel cellular behaviors. For example, in recent years, numerous synthetic gene circuits have been developed that respond to small‐molecular drugs,^[^ ^1^, ^2^, ^3^ ^]^ compounds naturally present in fruits or vegetables,^[^ ^4^, ^5^, ^6^ ^]^ or supplements added during food processing,^[^ ^7^, ^8^, ^9^ ^]^ as well as physical stimuli^[^ ^10^, ^11^, ^12^, ^13^ ^]^ to drive therapeutic protein expression in mammalian cells in a dose‐ and spatiotemporally‐controlled manner.^[^ ^14^ ^]^ Small‐molecule‐regulated transgene switches offer many advantages, such as easy administration, good reversibility, and scalability.^[^ ^5^, ^15^, ^16^ ^]^ Many of the reported gene circuits are based on bacterial transcription factors (TFs) containing a small molecule sensing domain and a DNA‐binding domain. While each bacterial TF exhibits a high degree of specificity toward single molecule inducers or groups of analogs, the overall collection of currently available bacterial‐derived gene switches respond to a diverse library of small molecules, enabling the assembly of higher‐order functions through orthogonal combinations.^[^ ^5^, ^7^, ^15^ ^]^ However, owing to their foreign nature, bacterial components of these switches may trigger immune responses in mammalian hosts.^[^ ^17^, ^18^ ^]^

To expand the range of compounds capable of regulating human‐derived gene switches, we focused on inducers of reactive oxygen species (ROS), such as peroxides, superoxide, hydroxyl radical, and singlet oxygen, which can be generated as by‐products of oxygen metabolism or during endogenous enzymatic reactions in mitochondria.^[^ ^19^ ^]^ Under normal physiological conditions, ROS play essential roles in defense against pathogens, cell growth, and redox signaling by regulating gene expression and cellular responses to environmental stimuli.^[^ ^20^ ^]^ However, excessive ROS production causes oxidative stress that damages cellular components, such as lipids, proteins, and DNA.^[^ ^21^ ^]^ To control ROS levels, mammals have evolved a native ROS biosensing system consisting of nuclear factor erythroid 2‐related factor 2 (NRF2) and Kelch‐like ECH‐associated protein 1 (KEAP1). To maintain homeostasis, KEAP1 retains NRF2 in the cytoplasm and targets it for ubiquitination by the Cullin3 KEAP1 E3 ligase, resulting in proteasome‐dependent degradation. Upon exposure to increased levels of ROS, NRF2 bypasses KEAP1 and translocates into the nucleus, where it binds to DNA sequences containing the antioxidant response element (ARE) and drives coordinated downstream expression of a battery of genes encoding antioxidant‐related proteins.^[^ ^21^, ^22^ ^]^

We previously showed that non‐toxic levels of ROS generated by direct current (DC)‐actuated regulation technology (DART) could be utilized to enable direct electrode‐mediated, time‐ and voltage‐dependent transgene expression in human cells using DC from a battery.^[^ ^12^ ^]^ The DART system employs a KEAP1‐derived ROS biosensor to reversibly fine‐tune NRF2‐mediated activation of synthetic promoters (P_DART_) containing AREs. In the present work, we aimed to examine whether a KEAF1‐NRF2 gene switch could be controlled by small‐molecule inducers generating low, non‐toxic levels of ROS. This would open up the possibility of using a broad range of structurally diverse compounds as inducers, including probes, markers, inhibitors, cofactors, etc., working in biological systems,^[^ ^23^, ^24^ ^]^ nutritional supplements, food preservatives, additives, and flavorings used in the food industry,^[^ ^25^ ^]^ and small‐molecule medicinal drugs.^[^ ^26^ ^]^ A key advantage of this approach is that an ROS scavenger, such as FDA‐approved *N*‐acetyl‐*L*‐cysteine (NAC),^[^ ^27^ ^]^ could be used to turn the gene switch off by decreasing the ROS levels in an emergency.

Here, we designed a generalized synthetic gene‐switch platform, named ROS_SENSE_, that responds to a broad range of ROS‐generating compounds to drive transgene expression by ectopically overexpressing KEAP1 and NRF2 to hypersensitize mammalian cells to ROS. We employed an ARE8‐containing promoter containing eight‐tandem ARE element repeats, as ARE8 is more sensitive to chemical‐induced ROS levels^[^ ^28^ ^]^ than the DART promoters used for sensing electrostimulated ROS.^[^ ^12^ ^]^ As a proof‐of‐concept, we constructed ROS_SENSE_‐engineered monoclonal cells stably expressing the KEAP1‐NRF2‐ARE8 system linked to insulin expression. When these cells were intraperitoneally implanted in mice with experimental type‐1 diabetes (T1D), intraperitoneal injection of a variety of chemicals regulated the production of sufficient insulin to maintain normoglycemia and reduce postprandial glycaemic excursions in the mice.

## 2 Results

### 2.1 Design and Characterization of a REM‐Inducible Transgene Expression System

**Figure 1:**
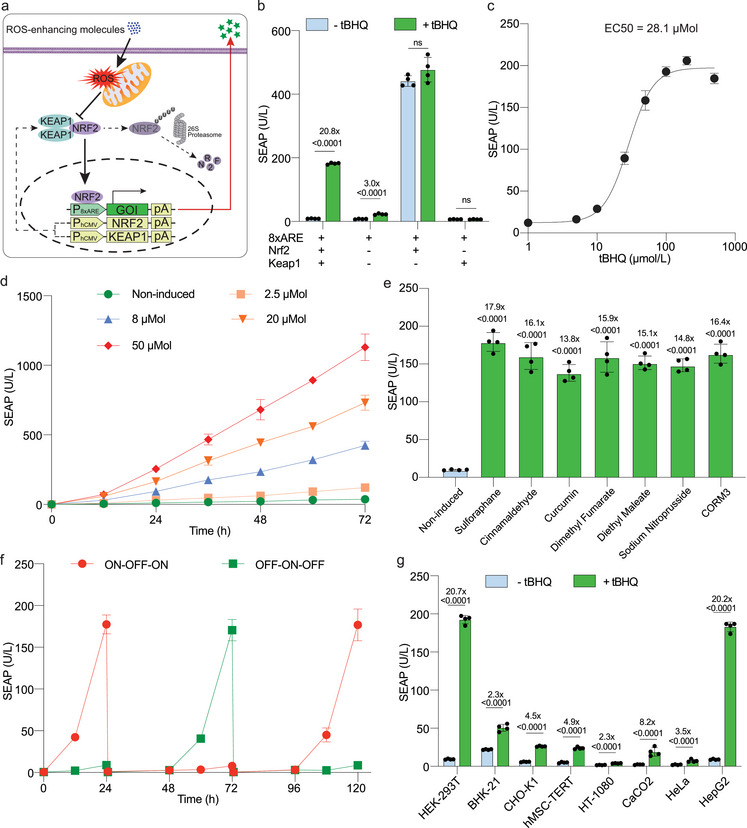
Design and validation of the ROS‐mediated transgene expression system in mammalian cells. a) Schematic illustration of the synthetic gene circuit based on the NRF2/KEAP1 signaling cascade. When intracellular ROS levels are low, ectopically expressed NRF2 and KEAP1 associate in the cytoplasm, where NRF2 undergoes continuous ubiquitination by KEAP1, leading to its transportation to the 26S proteasome for degradation. Upon exposure to ROS‐enhancing molecules (e.g., tBHQ), elevated ROS levels disrupt the KEAP1/NRF2 complex, thereby allowing NRF2 translocation to the nucleus, where it binds to ARE elements in a synthetic promoter, triggering the activation of the gene of interest (GOI). b) SEAP production in HEK‐293T cells transiently transfected with ARE reporter (pJH1227, O_ARE8_‐SEAP) alone or together with NRF2 (pJH1003) or/and KEAP1 (pJH1004). c) Dose‐response curve of transiently transfected HEK‐293T cells treated with tBHQ, indicating the EC50 value. d) Dose‐dependent SEAP production over a 72‐h period in engineered cells exposed to tBHQ at the specified concentrations. e) Extracellular SEAP levels following 24 h of treatment with the ROS‐enhancing compounds sulforaphane (10 µmol L^−1^), cinnamaldehyde (50 µmol L^−1^), curcumin (50 µmol L^−1^), dimethyl fumarate (100 µmol L^−1^), diethyl maleate (100 µmol L^−1^), sodium nitroprusside (400 µmol L^−1^) and CORM3 (500 µmol L^−1^). f) Reversibility of the synthetic transgene switch. Engineered cells were alternately cultured for 24 h intervals in tBHQ‐free medium (OFF) or medium containing 40 µmol L^−1^ of tBHQ (ON). The culture medium was replaced and the cell density was readjusted every 24 h. g) SEAP production by various mammalian cell lines transiently transfected with the ROS_SENSE_ system. The SEAP levels were quantified in the culture supernatants. U/L: unit per liter; In (b,g), all treatment groups were induced with 40 µmol L^−1^ tBHQ (+ tBHQ), and the non‐treated groups received an equivalent amount of vehicle (‐ tBHQ). In (c,d), cells were treated with the indicated concentration of tBHQ. All data are means ± SD; n = 4. *P* values in (e) and (g) were calculated versus the corresponding non‐induced control.

To build a transcriptional regulatory switch responsive to non‐toxic concentrations of ROS‐enhancing small molecules (REMs) in mammalian cells, we constitutively expressed NRF2 (pJH1003, P_hCMV_‐NRF2‐pA) and KEAP1 (pJH1004, P_hCMV_‐KEAP1‐pA), along with a reporter gene encoding a human placental secreted alkaline phosphatase (SEAP) driven by a synthetic promoter containing ARE (**Figure** **1**a). First, we screened different promoters containing up to eight tandem repeats of AREs (Figure S1a, Supporting Information) and found that using from 5 to 8 repeats (ARE5 to ARE8) significantly increased the fold‐induction of SEAP production in cells treated with the classical ROS inducer tert‐butylhydroquinone (tBHQ), which is widely used as a food preservative.^[^ ^22^, ^28^ ^]^ This is consistent with our previous finding that a promoter containing 4 repeats (ARE4) was highly responsive to DC induced ROS, but much less sensitive to small‐molecule inducers of ROS.^[^ ^12^ ^]^ Constitutively expressing both NRF2 and KEAP1 was crucial for switch functionality, as skipping either of the proteins abolished tBHQ responsiveness, while skipping both greatly decreased the fold‐induction (from 20x to 3x), as well as the SEAP production level (Figure 1b). We also observed that the mass ratio of the three plasmids influences the switch performance, with the highest reporter fold‐induction obtained at a ratio of 1:5:2 for NRF2 (pJH1003), KEAP1 (pJH1004) and ARE8 (pJH1227), respectively (Figure S1b,c, Supporting Information). Furthermore, we tested the impact of using different promoters, P_SV40_ (simian virus 40 promoter), P_mPGK_ (murine phosphoglycerate kinase promoter), P_hEF1a_ (human elongation factor‐1 alpha promoter) and P_hCMV_ (human cytomegalovirus immediate early promoter), to express NRF2 and KEAP1 on the switch functionality (Figure S1d–h, Supporting Information). The most favorable response to tBHQ treatment occurred when both proteins were expressed from a P_hCMV_ promoter (Figure S1d–h, Supporting Information).

Dose‐response studies with cultures of HEK‐293T cells transiently transfected with the best‐performing switch construct revealed that tBHQ induced SEAP expression with a half‐maximal effective concentration (EC50) of 28.1 µm (Figure 1c; Figure S2a, Supporting Information). Furthermore, the production level of SEAP increased linearly over time at different tBHQ concentrations, with increased activity ratios of 20‐ or 30‐fold after 3 days of treatment with 20 or 50 µm tBHQ, respectively, relative to non‐treated cell cultures (Figure 1d). A fluorescence‐based quantification assay revealed a dose‐dependent increase in intracellular ROS levels of up to 2.5‐fold at 1 h after tBHQ treatment (Figure S2b,c, Supporting Information). Furthermore, cells treated with the ROS scavenger NAC exhibited a substantial reduction in SEAP expression (Figure S2d,e, Supporting Information), confirming the ROS‐mediated nature of the chemically induced SEAP expression.

Next we assessed the responsiveness of our optimized ROS‐mediated gene switch to a variety of ROS‐enhancing small molecules potentially applicable in therapeutic settings, including sulforaphane,^[^ ^22^, ^28^ ^]^ an anticancer compound found in cruciferous vegetables, along with plant‐derived molecules cinnamaldehyde and curcumin, commonly used as food flavorings.^[^ ^22^, ^29^ ^]^ All three compounds robustly activated SEAP expression (Figure 1e). We also examined dimethyl fumarate, an FDA‐approved drug for psoriasis and multiple sclerosis treatment,^[^ ^30^ ^]^ and diethyl maleate, a pharmaceutical intermediate.^[^ ^22^, ^31^ ^]^ Both compounds triggered reporter expression, with fold‐inductions of 15.9x and 15.1x, respectively (Figure 1e). Finally, to examine the effect of gaseous signaling molecules, we targeted nitric oxide (NO) and carbon monoxide (CO), which are critical second messengers in mammalian signaling transduction pathways, using sodium nitroprusside and CORM3 as NO and CO donors,^[^ ^32^, ^33^ ^]^ respectively. Both compounds induced SEAP expression significantly above background levels (Figure 1e). The elevated ROS levels in the engineered cells had a negligible impact on cell viability, and are similar to those observed in our DC‐stimulated ROS sensing system, DART (Figure S3, Supporting Information), suggesting that the tested chemicals would also exhibit minimal interference with endogenous cellular physiology at the concentrations used for switch activation.^[^ ^12^ ^]^ These outcomes underscore the inducibility of the system across a diverse spectrum of clinically compatible ROS‐enhancing molecules. We further examined various additional compounds, including traditional ROS inducers such as oltipraz^[^ ^22^ ^]^ and hydrogen peroxide,^[^ ^22^, ^34^ ^]^ alongside molecules with documented antioxidant properties such as aspirin,^[^ ^35^ ^]^ salicylic acid,^[^ ^36^ ^]^ and vitamin C^[^ ^37^ ^]^ (Figure S4a–i, Supporting Information). In addition, we assessed the impact of conventional small‐molecule inducers, doxycycline,^[^ ^38^ ^]^ rapamycin,^[^ ^39^ ^]^ abscisic acid,^[^ ^40^ ^]^ and vanillic acid^[^ ^41^ ^]^ (Figure S4j–q, Supporting Information). None of these compounds induced SEAP expression at moderate concentrations, suggesting some degree of specificity of the developed ROS‐mediated synthetic gene circuit, and supporting its orthogonality with respect to these conventional inducers.

To test the reversibility of the system, engineered cells were iteratively/cyclically incubated with tBHQ or vehicle (DMSO) at intervals of 24 h. A robust reversibility performance was observed, with similar activation or repression patterns during ON‐OFF‐ON and OFF–ON–OFF cycles (Figure 1f). Furthermore, the generality of the system was evaluated in a variety of rodent and human cell lines in transient transfection experiments (Figure 1g). Although variations in expression levels and induction folds were observed across different cell types following exposure to tBHQ, the functionality of the system was validated in all tested cell lines, including human mesenchymal stem cell‐derived hMSC‐TERT and liver‐derived HepG2 cells, suggesting a broad range of compatibility and applicability of the ROS_SENSE_ system (Figure 1g; Figure S5, Supporting Information). Considering the fold‐inductions, and basal and maximal expression levels, we opted to proceed with HEK‐293T cells for subsequent experiments.

### 2.2 Generation and Characterization of a ROS_SENSE_‐Modified Monoclonal Cell Line

**Figure 2:**
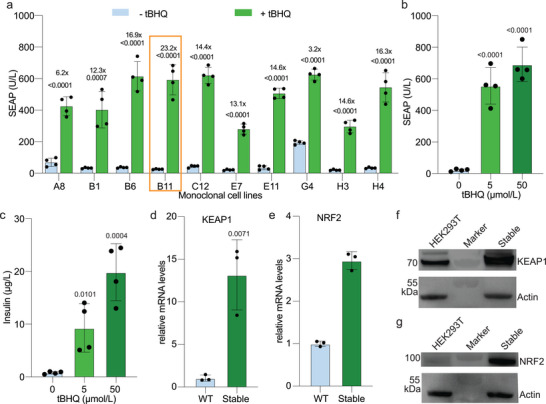
Screening and validation of monoclonal stable cell lines. a) Evaluation of the top ten clones obtained from an initial screening. SEAP levels were quantified in the culture supernatant after induction for 24 h. The most promising monoclonal cell line, designated ROS_SENSE_‐INS and highlighted in orange, was selected for follow‐up experiments. b,c) SEAP (b) and insulin (c) produced by ROS_SENSE_‐INS cell line in the culture supernatant after tBHQ induction for 24 h at the indicated concentrations. d,e) Relative mRNA levels of d) KEAP1 and e) NRF2 in wild‐type cells (WT) and ROS_SENSE_‐INS cells, analyzed by quantitative real‐time PCR. f,g) Western blots of KEAP1 (f) and NRF2 (g) proteins in wild‐type cells (WT) and selected stably transfected cells. The same amount of cell lysate was loaded on the gel and the housekeeping protein actin was used as a loading control. The experiments were repeated independently with similar results in b‐h. WT: wild type. Stable: ROS_SENSE_‐INS cell line. All data are presented as means ± SD; in (a–c), *n* = 4; in (d,e), *n* = 3. *P* values were calculated for the differences between induced and non‐induced control groups.

As a proof‐of‐concept study in vivo, we decided to target T1D, a highly prevalent chronic disease.^[^ ^42^ ^]^ Initially, we used the Sleeping Beauty transposase system^[^ ^43^ ^]^ to establish a monoclonal cell line in which the switch components for regulating the expression of both mouse insulin and SEAP (Figure S6a, Supporting Information) were stably integrated into the genome of HEK‐293T cells. To select a cell clone with high transgene induction, we profiled 96 clones in a primary screening (Figure S6b,c, Supporting Information) and the top performers were assessed in a secondary screening (**Figure** **2**a). The best‐in‐class monoclonal cell line, ROS_SENSE_‐INS, showed high tBHQ‐induced SEAP expression and insulin production (Figure 2b,c), with slightly higher fold‐induction and much higher SEAP levels than transiently transfected cells after the same 24 h tBHQ treatment (Figure 1b,c). A cell‐based insulin activity assay was performed in engineered HEK‐293T cells ectopically expressing an insulin receptor to confirm that the insulin produced by ROS_SENSE_‐INS cells is physiologically active (Figure S6d,e, Supporting Information). We also performed quantitative PCR (qPCR) and western blotting to compare NRF2 and KEAP1 transcript and protein levels between ROS_SENSE_‐INS and parental cells. The transcriptional levels of both genes were significantly upregulated in ROS_SENSE_‐INS cells (Figure 2d,e), as were the protein levels (Figure 2f,g), when compared to wild‐type cells.

**Figure 3:**
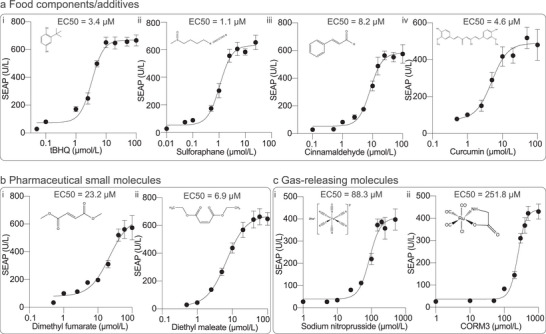
Dose‐response curves of the stable cell line treated with various ROS‐enhancing molecules. a) Responsiveness of ROS_SENSE_‐INS cells to various food components/additives: i) tert‐Butylhydroquinone (tBHQ), ii) sulforaphane, iii) cinnamaldehyde and iv. curcumin. b) Responsiveness to pharmaceutical molecules: i) dimethyl fumarate and ii) diethyl maleate. c) Responsiveness to gas‐releasing molecules: i) sodium nitroprusside and ii) CORM3. The EC50 value of each compound is indicated in each panel. All data are presented as means ± SD; *n* = 4.

Next, we obtained dose‐response curves for each of the candidate small molecules previously identified as inducing SEAP expression in transiently transfected cultures. Notably, the ROS_SENSE_‐INS cells exhibited increased sensitivity to the food preservative tBHQ, with an EC50 of 3.4 µM (**Figure** **3**a), which is 8.5‐fold lower than that obtained in transiently transfected cells (Figure 1c). Similarly, the cells exhibited elevated sensitivity to sulforaphane, with an EC50 of 1.1 µm, and slightly lower sensitivity to cinnamaldehyde and curcumin, with EC50 values of 8.2 and 4.6 µm, respectively (Figure 3a). Diethyl maleate and dimethyl fumarate showed EC50 values of 23.2 and 6.9 µMol, respectively (Figure 3b). Finally, the NO and CO donors also showed dose‐dependent induction of ROS_SENSE_‐INS stable cells, albeit with markedly higher EC50 values (EC50 of 88.3 and 251.8 µm, respectively) relative to the other compounds (Figure 3c). We also observed a dose‐dependent increase in intracellular ROS levels of ROS_SENSE_‐INS cells upon exposure to all these small molecules (Figure S7, Supporting Information), strongly supporting the ROS‐mediated nature of the transgene regulation system. Importantly, none of the tested compounds had a substantial impact on cell viability within the relevant concentration ranges (Figure S8, Supporting Information), nor did they influence cell growth kinetics or overall recombinant protein production capacity at the respective EC50 concentrations (Figure S9, Supporting Information).

### 2.3 Expression Kinetics and Reversibility of ROS_SENSE_‐INS Cells Treated with Various REMs

**Figure 4:**
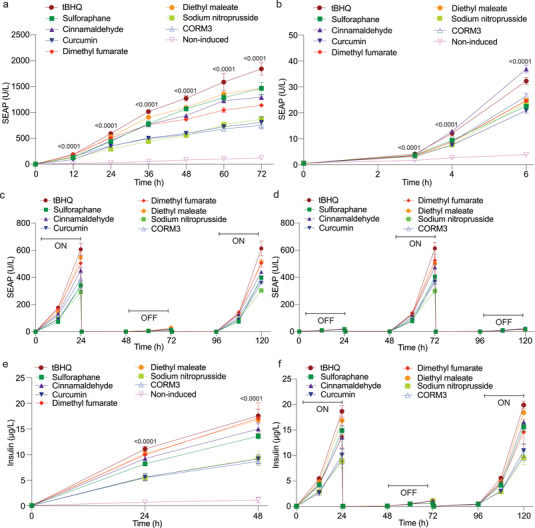
Expression kinetics and reversibility of the monoclonal cell line exposed to ROS‐enhancing molecules. a) Time‐dependent induction of SEAP production over 72 h in ROS_SENSE_‐INS cells exposed to the indicated chemicals. b) Time‐dependent SEAP production within 6 h in ROS_SENSE_‐INS cells after exposure to the indicated chemicals. c,d) Reversibility of SEAP production in ROS_SENSE_‐INS cells. The cells were alternately cultured in medium without REMs (OFF) or with the indicated REMs (ON) in an c) ON–OFF–ON or d) OFF‐ON‐OFF cycle with 24 h intervals. The culture medium was replaced, and the cell density was re‐adjusted every 24 h. e) Time‐dependent insulin production over 48 h in ROS_SENSE_‐INS cells exposed to the indicated compounds. f) Reversibility of insulin production in ROS_SENSE_‐INS cells. The cells were alternately cultured in medium containing ROS‐enhancing compounds (ON) or in standard medium (OFF) in an ON–OFF–ON cycle with 24 h intervals. The compounds were added at twice their EC50 concentrations based on Figure 3. SEAP and insulin were measured in the culture supernatants. All data are presented as means ± SD; *n* = 4. The *p*‐values indicate the significance of differences in the mean values versus the non‐induced group, and the *p*‐values for all data points after induction are <0.0001.

Next, we characterized the SEAP expression kinetics of ROS_SENSE_‐INS cells following exposure to the ROS‐enhancing molecules. We observed SEAP accumulation over the course of three days for all compounds. Protein production varied, likely reflecting not only the induction capability of each compound but also differences in stability in the culture medium (**Figure** **4**a). Significant differences between the treatment groups and non‐treated cells appeared shortly after introducing the ROS‐enhancing molecules, with a twofold upregulation within 3 h of tBHQ treatment (Figure 4b). Furthermore, the ROS_SENSE_‐INS cells exhibited excellent reversibility of SEAP expression for all compounds in response to ON–OFF–ON (Figure 4c) or OFF–ON–OFF cycles (Figure 4d), achieved by changing the culture medium at 24‐h intervals. Importantly, similar expression kinetic patterns were obtained for insulin in response to the various inducers (Figure 4e), and the insulin response to cycles of ON‐OFF‐ON at 24‐h intervals showed excellent reversibility (Figure 4f).

### 2.4 REM‐Induced Insulin Expression for the Treatment of T1D

**Figure 5:**
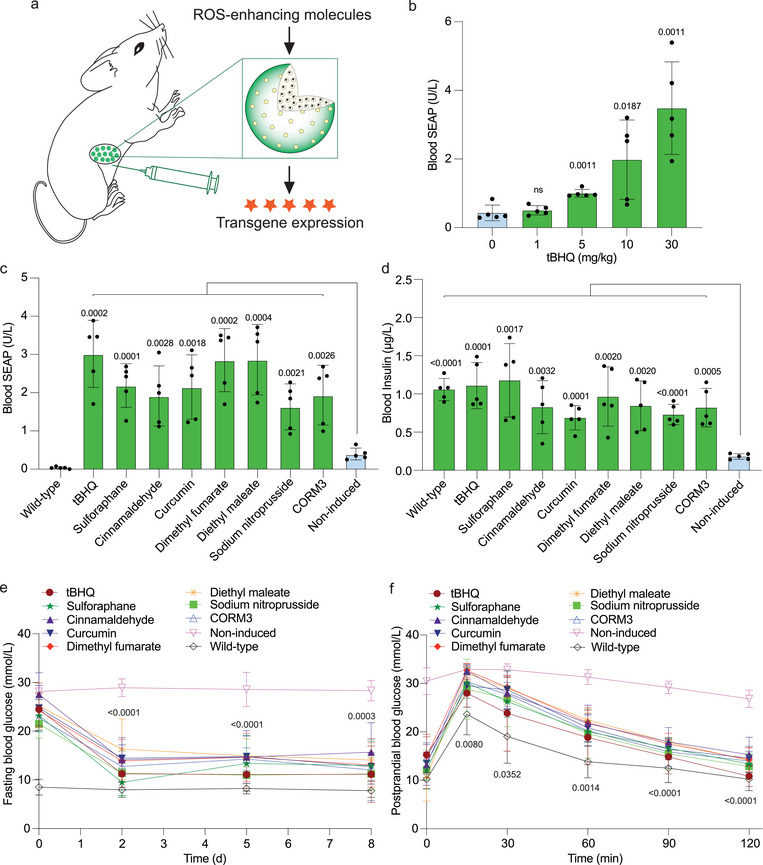
Validation of the ROS_SENSE_ system to regulate transgene expression in type‐1 diabetic mice. a) Schematic model showing encapsulated engineered cells implanted in the mice. The encapsulated cells are implanted intraperitoneally and chemical inductions were performed locally. b) Serum SEAP levels in wild‐type mice following intraperitoneal administration of the indicated dosages of tBHQ. c) Serum SEAP levels in wild‐type mice following intraperitoneal administration of the indicated ROS‐enhancing molecules. d) Serum insulin levels in T1D mice following intraperitoneal administration of the indicated ROS‐enhancing molecules. e) Fasting glycemia levels were recorded over one week of treatment. Mice injected with the encapsulated ROS_SENSE_‐INS cells were untreated (Non‐induced group) or treated with the indicated compounds twice per day. Wild‐type mice without any implantation and treatment were used as the control (Wild‐type group). f) Intraperitoneal glucose tolerance test (GTT). GTT was performed on day 3 after implantation of microencapsulated cells and after fasting for 8 h in wild‐type mice, non‐induced T1D mice and T1D mice treated with the indicated compounds. In (c–f), doses of tert‐butylhydroquinone (tBHQ), sulforaphane, cinnamaldehyde, curcumin, dimethyl fumarate, diethyl maleate, sodium nitroprusside, and CORM3 of 30, 5, 50, 100, 100, 5, 5, and 50 mg kg^−1^/day, respectively, were administered intraperitoneally. All data are means ± SD; n = 5; the values were normalized to the wild‐type group. The *p*‐values indicate the significance of differences in the mean values: in (b), treatment groups versus non‐induced group; in (c) and (d), non‐induced group versus the indicated groups; in (e) and (f), the tert‐butylhydroquinone (tBHQ) group versus non‐induced group.

To investigate the in vivo performance of the ROS_SENSE_‐INS system, we micro‐encapsulated the stable cells in FDA‐licensed semi‐permeable alginate beads, which provide protection from the host immune system, and at the same time enable free diffusion of nutrients and therapeutic protein. Pre‐implantation, the microencapsulated ROS_SENSE_‐INS cells showed dose‐dependent SEAP (Figure S10a, Supporting Information) and insulin (Figure S10b, Supporting Information) production in response to tBHQ. To validate the performance in vivo, we implanted the encapsulated engineered cells intraperitoneally (i.p.) in wild‐type mice, which were then treated with different concentrations of tBHQ (**Figure** **5**a). Analysis of blood samples showed tBHQ‐dose‐dependent SEAP expression in mice (Figure 5b), confirming precise in vivo regulation of the ROS_SENSE_‐INS system by tBHQ.

Next, we injected (i.p.) the alginate‐encapsulated ROS_SENSE_‐INS cells into experimental T1D mice to assess the therapeutic protein expression profile. We first confirmed that the ROS_SENSE_ system was induced by ROS‐enhancing compounds by profiling SEAP expression levels in the blood (Figure 5c). We further confirmed that REM‐treated T1D mice showed significantly higher blood insulin levels than non‐treated T1D mice (Figure 5d). All compounds could attenuate glycemia within two days and maintain glucose homeostasis throughout treatment (Figure 5e). An intraperitoneal glucose tolerance test (GTT) revealed that REM‐induced insulin production and release could restore normoglycemia within two hours, while the non‐induced control group remained hyperglycemic (Figure 5f). This confirms that the ROS_SENSE_‐INS system is functional in vivo and can be precisely regulated by various compounds for therapeutic protein expression.

## 3 Discussion

The ROS_SENSE_ system is a platform for controlling therapeutic protein expression in mammalian cells in response to compounds that generate low, non‐toxic levels of ROS. Multiple species of ROS exist, and their induction can be attributed to the pro‐oxidant properties of numerous chemicals during various enzymatic processes within mammalian cells. Therefore, our ROS‐sensing system exhibits versatility, as it can be driven by a broad range of structurally unrelated chemicals, including medicinal drugs, metabolism‐related compounds, and food additives, opening up many opportunities for controlling cell‐based gene therapies for disease management. To build hypersensitive gene switches responsive to minute amounts of ROS, we ectopically overexpressed the proteins KEAP1 and NRF2, which are part of an endogenous transcriptional program activated during periods of oxidative stress to re‐establish redox homeostasis by upregulating genes with antioxidant potential (downstream of ARE‐containing promoters). We showed that the expression levels of NRF2 and KEAP1 impact the overall performance of the ROS_SENSE_ system. Importantly, non‐toxic levels of ROS‐enhancing compounds showed an insignificant impact on key viability parameters, including growth kinetics and recombinant protein production capacity, of parental cells devoid of ROS_SENSE_. This is consistent with a previous study showing that non‐toxic levels of ROS generated by DC had no significant effect on the cellular transcriptome, as determined by comprehensive high‐throughput RNA‐sequencing analysis.^[^ ^12^ ^]^ While we did not conduct RNA‐sequencing in the present work to assess whether the chemical treatments influenced the overarching cellular physiology, the ROS levels and cell viability in the chemical‐induced system were similar to those in our DC‐stimulated DART system,^[^ ^12^ ^]^ suggesting that the impact of the chemical‐induced ROS generation on cellular dynamics is likely to be minimal, as in the case of the DC‐stimulated ROS generation, notwithstanding that the pattern of ROS species production may not be the same. These results suggest that native cells, devoid of ectopic KEAP1/NFR2 expression and lacking highly optimized ARE‐based promoters, remain insensitive to REMs at levels triggering transgene expression in ROS_SENSE_ cells. The best fold‐inductions were obtained when expressing both genes from the strong human cytomegalovirus promoter (P_hCMV_) and using a mass ratio of 1:5 between the *NRF2*‐ and *KEAP1*‐encoding plasmids. In the endogenous NRF2‐KEAP1 antioxidant program, one NRF2 protein molecule physically interacts with a homodimeric KEAP1, so that the stoichiometry is 1:2.^[^ ^21^, ^22^ ^]^ In the ROS_SENSE_ system, we found that expression of more KEAP1 helps to suppress leaky expression in the basal state resulting from the hyper‐transactivation activity of NRF2. Furthermore, ARE8, having eight‐tandem ARE repeats without any spacer, was found to be the best promoter configuration to control the expression of the gene of interest in response to different ROS‐enhancing molecules. Therefore, by fine‐tuning promoters and the molar ratios of plasmids, we were able to develop tailored approaches to minimize the heterogeneity of therapeutic protein expression, which is important to enhance the predictability and consistency of therapeutic outcomes.

In the landscape of gene‐ and cell‐based therapies, small molecules can be highly effective and practical as input signals for controlling in vivo production of biopharmaceuticals. To explore their efficacy, we applied our multi‐purpose human‐derived ROS_SENSE_ switch in a translational proof‐of‐principle study focused on treating T1D. Blood‐glucose monitoring and glucose‐tolerance tests in T1D mice showed that the ROS_SENSE_ system provided sufficient insulin production to maintain daily glycemia and attenuate postprandial glycemic excursions for all the ROS‐enhancing compounds under study. Thus, we believe that this multiple‐chemical‐triggered transgene switch will enable the integration of various molecular inducers into one cell‐based gene therapy system. Furthermore, it holds promise for the development of combination therapies by coupling classical small‐molecule therapies with trigger‐induced production of biopharmaceuticals targeting the same medical condition as the trigger compound. For example, it could be foreseen that rapalogs, clinically licensed for the treatment of leukemia, could serve their original therapeutic purpose, while also controlling the expression of companion biopharmaceuticals such as cytokines in a cell‐therapy scenario.^[^ ^44^, ^45^ ^]^ It should be noted that although the ROS‐sensitive gene switches are activated by minute concentrations of ROS‐enhancing molecules, typically within the low micromolar range, these concentrations are significantly higher than those found in processed foodstuffs, so ROS_SENSE_ cells are expected to remain unresponsive to dietary intake. Overall, our human‐based gene switch exhibits robust reversibility and tight regulation, with low basal expression in the absence of ROS‐enhancing molecules and provides a wide dynamic control range for most of the compounds tested, making it a control platform with wide appeal and broad potential applicability in basic, applied and clinical research.

Control of therapeutic protein expression by means of external triggers represents a revolutionary avenue in medical research, offering compelling advantages for clinical translation. The capacity to finely regulate therapeutic protein expression in response to external signals enables a flexible and customizable approach that allows optimization of therapeutic efficacy while minimizing unintended side effects. This dynamic control provides a basis for constructing personalized treatment strategies tailored to each patient's needs, where delivery of a therapeutic protein can be activated or deactivated as needed, akin to a light switch. Such precision holds particular promise for treating chronic conditions and for targeted cancer therapies. However, the success of this approach in clinical settings hinges on further key developments. In particular, careful refinement of trigger‐responsive systems is essential to optimize specificity, reliability, and efficiency. Rigorous preclinical validation, including extensive testing in laboratory and animal models, is imperative to ensure the safety and efficacy of these systems before advancing to human trials. Standardized protocols for the integration of controlled therapeutic protein expression into existing clinical practices are also needed to permit a smooth transition from laboratory to bedside. Addressing these challenges will be critical not only to unlock the full translational potential of this technology, but also to lay the groundwork for regulatory approval.^[^ ^14^ ^]^ In summary, the key advantages of synthetic‐biology‐controlled therapeutic protein delivery lie in its precision, adaptability, and safety, which are expected to contribute to enhanced therapeutic outcomes.

## 4 Experimental Section

### Plasmid Construction

A comprehensive list of all plasmids is provided in Table S1 (Supporting Information). Polymerase chain reaction (PCR) was performed to amplify the fragments using Q5 high‐fidelity polymerase (M0491). The vectors were constructed either by using restriction endonucleases digestion followed by T4 ligation or by Gibson assembly. The enzymes were from New England Biolabs (Ipswich, MA, USA). All the plasmids were propagated in XL10‐gold K12 *E. coli* (Stratagene). All plasmid sequences were verified by Microsynth AG (Balgach, Switzerland).

### Main Chemicals and Reagents

Streptozotocin (STZ, cat. no. S0130‐1G), Tween 80 (cat. no. P1754‐500ML), tBHQ (cat. no. 112941–5G), sodium nitroprusside (cat. no. 71778‐25G), diethyl maleate (cat. no. W505005), dimethyl fumarate (cat. no. 242926‐25G), cinnamaldehyde (cat. no. W228613‐100G‐K), curcumin (cat. no. C1386‐5G), sulforaphane (cat. no. 574215–25MG), CORM3 (cat. no. SML0496‐50MG), ML171 (cat. no. 492002–10MG), N‐acetyl‐L‐cysteine (cat. no. A9165‐5G), D‐glucose (cat. no. G7528‐250G), doxycycline (cat. no. D9891‐1G), rapamycin (cat. no. 553211‐1MG), abscisic acid (cat. no. 90769‐25MG), vanillic acid (cat. no. H36001‐25G) and recombinant human insulin (catalog no. PHR8925) were purchased from Sigma Aldrich (Buchs, Switzerland). Puromycin (ant‐pr‐1), zeocin (ant‐zn‐05), and blasticidin (ant‐bl‐05) were from InvivoGen (Toulouse, France). Alginate (w/v, 1.8%; Na‐alginate, cat. no. 11061528) was from Buechi (Buechi Labortechnik AG, Switzerland), and PLL 2000 (w/v, 0.05%; PLL 2000: cat. no. 25988‐63‐0) was from Alamanda (Alamanda Polymers Inc., USA).

### Cell Culture and Transfection

Human embryonic kidney cells (HEK‐293T, ATCC: CRL‐11268), baby hamster kidney cells (BHK‐21, ATCC: CCL‐10), Chinese hamster ovary cells (CHO‐K1, ATCC: CCL‐61), human telomerase‐immortalized mesenchymal stem cells (hMSC‐TERT, RRID:CVCL_Z015), human fibrosarcoma cells (HT‐1080, ATCC: CCL‐121), human colorectal adenocarcinoma cell line (Caco‐2, ATCC: HTB‐37), human cervical adenocarcinoma cells (HeLa, ATCC: CCL‐2), and human liver cancer cell line (Hep G2, ATCC: CRL‐11997) were cultivated in Dulbecco's modified Eagle's medium (DMEM, cat. no. 52100–39, Thermo Fisher Scientific) supplemented with 10% fetal bovine serum (FBS, cat. no. F7524, lot no. 022M3395, Sigma‐Aldrich) and 1% (v/v) streptomycin/penicillin (cat. no. L0022, Biowest) under a humidified atmosphere containing 5% CO_2_ at 37 °C. Proline (100 mm) was added in the medium for CHO‐K1 only. For transfection, 15 000 cells were seeded per well in a 96‐well plate (cat. no. 3599, Corning Inc. Life Sciences), incubated overnight and transfected for more than 6 h by addition of 20 µl FBS‐free DMEM mixture containing 125 ng of DNA per well and polyethyleneimine (PEI, 24765‐2, Polysciences) in a ratio of 1:4. The culture medium was then replaced by complete medium with or without inducers. Cells were continuously cultivated for >24 h before the supernatant was collected for quantitative analysis of secreted reporter. Tested cells were typically characterized after one passage in the same culture medium in order to ensure they maintained the desired characteristics and functionality. This post‐passage characterization involved assessing a wide range of cellular features, such as morphology, viability, growth rate, and expression of targeting genes at the transcriptional and translational levels, as well as microscopy, other imaging, and flow cytometry.

### Monoclonal Stable Cell Line Generation

Stably transfected HEK‐293T cells were generated using the Sleeping Beauty transposon system (Mátés et al. 2009). 50 000 cells per well were seeded into a 24‐well plate (cat. no. 3524, Corning Inc. Life Sciences) for 24 h. Then the cells were co‐transfected with pJH1101(200 ng), pJH1102 (1000 ng), pJH1237 (400 ng) and pJH42 (160 ng), and incubated for 24 h. The transfected cells were transferred into a 10 cm diameter dish (Greiner Bio‐one, cat. no. 664160) and incubated for 10 days in the presence of 100 µg ml^−1^ zeocin, 10 µg ml^−1^ blasticidin, and 2 µg ml^−1^ puromycin for antibiotic selection. The monoclonal cell lines were screened by the addition of tBHQ and the best‐in‐class cell line was selected for in vitro and in vivo studies.

### SEAP Quantification

Human placental SEAP reporter assay was performed to profile SEAP levels in cell culture supernatants. Briefly, 80 µl heat‐inactivated (30 min at 65 °C) cell culture supernatant was mixed with 80 µl 2x SEAP assay buffer (20 mm homoarginine, 1 mm MgCl_2_, 21% diethanolamine, pH 9.8). Next, 20 µL substrate solution (120 mm p‐nitrophenyl phosphate, cat. no. AC128860100, Thermo Fisher Scientific) in 2x SEAP buffer was added to each well. The absorbance at 405 nm was recorded by a plate reader (Tecan Group Ltd., Maennedorf, Switzerland) for 30 min at 37 °C and the SEAP levels were determined from a standard curve.

### Nano‐Luciferase Quantification

7.5 µl of supernatant from the cell culture medium was mixed with 7.5 µl working solution containing luciferase substrate and buffer in each well of into a black 384‐well plate (cat. no. CLS3658, Corning Inc. Life Sciences) according to the instructions provided with the Nano‐Glo Luciferase Assay Kit (cat. no. N1130, Promega). The luminescence was recorded by a plate reader (Tecan Group Ltd., Maennedorf, Switzerland).

### Cell Viability

Cells were incubated with resazurin (50 µg mL^−1^, cat. no. R7017, Sigma‐Aldrich) for 1 h. The fluorescence was recorded at 540/590 nm with a Tecan Infinite 200 PRO plate reader (Tecan Group AG, Switzerland).

### ROS Quantification

Cells were incubated with chemicals for 1 h at the indicated concentrations, washed with 300 µl phosphate‐buffered saline (PBS, cat. no. 14190‐094, Thermo Fisher Scientific), and placed in 50 µl FBS‐free DMEM medium containing 25 µmol/l 2′,7′‐dichlorofluorescein diacetate (DCFDA, cat. no. D6883, Sigma‐Aldrich) for 30 min. Then the cells were washed again with 30 µl PBS, and the ROS levels were quantified by fluorescence assay (485/535 nm, Tecan Infinite 200 PRO).

### Electrostimulation with DC

Measurement of DC‐induced ROS levels and viability assay of cells engineered with the previously developed electrogenetic interface, DART, were performed as previously described.^[^ ^12^ ^]^ Briefly, DART‐engineered HEK‐293T cells were cultured in a 24‐well plate for 24 h with 700 µL culture medium. DC was applied via platinized electrodes fixed on a customized lid^[^ ^12^ ^]^ for the indicated voltages and time periods using a DC power supply (KD3005P, KORAD). Half an hour later, ROS levels and cell viability were profiled.

### Chemical Structures

Chemical structures were drawn by ChemDraw software (V20.0.0.38, PerkinElmer).

### Insulin Quantification Bioassay

Quantification of insulin in cell cultures was performed using a cell culture‐based bioassay. Culture supernatant containing recombinant insulin was transferred to the insulin‐activity bioassay system, which consisted of HEK‐293T cells expressing insulin receptor (phIR), P_TRE_‐NanoLuc‐pA (pLeo1403), and Elk1‐TetR (Mkp37). Once triggered by insulin, the insulin receptor activated the MAPK signaling cascade that ultimately resulted in the expression of nano‐luciferase (NanoLuc).

### Effect of Chemicals on Cell Growth and Productivity

3.0 × 10^6^ HEK‐293T cells were transfected with 30 µg of pJH3 (P_hCMV_‐SEAP‐pA) in a 10 cm diameter dish (Greiner Bio‐one, cat. no. 664160) for 24 h. The cells were collected, resuspended, and evenly distributed into three 24‐well plates. The treatment groups were incubated with the indicated chemicals for 48 h. Samples were collected at the indicated time points to profile viable cell count and recombinant protein production.

### Western Blot

The cells were lysed by continuous agitation in RIPA buffer containing 150 mm NaCl, 50 mm Tris‐HCl 8.0, 1% Nodidet P‐40 (NP40), 0.5% sodium deoxycholate, 0.1% sodium dodecyl sulphate, 1 mm sodium orthovanadate, 1 mm NaF and protease inhibitors (Roche) at 4 °C. Then the lysate was centrifuged at 16000× g for 30 min at 4 °C and the supernatant was collected. The total protein concentration was quantified using a Pierce BCA Protein Assay Kit (cat. no. 23225, ThermoFisher). Then the samples were heated in 2x Laemmli buffer at 95 °C and subjected to SDS‐polyacrylamide gel electrophoresis. The samples in the gel were transferred to a polyvinylidene fluoride membrane (cat. no. 88518, ThermoFisher) in a transfer buffer containing 25 mm Tris, 190 mm glycine, and 20% methanol. Then the membranes were blocked with 5% nonfat milk at room temperature for 1 h, and incubated with primary antibody overnight at 4 °C, followed by incubation with secondary antibody at room temperature for 1 h. Blots were profiled using a chemiluminescence detection system (FusionPulse TS, cat. no. 121172301, v5.12a) after adding chemiluminescent substrate (Pierce ECL Western Blotting Substrate, cat. no. 32106, ThermoFisher).

### Quantitative PCR

The stably transfected cells were cultivated for 24 h before mRNA sample extraction. Total mRNA was extracted using a Quick‐RNA Miniprep Kit (Zymo Research, cat. no. R1054), and quantified by a NanoDrop 2000 (ThermoFisher). The cDNA library was prepared with a High‐Capacity cDNA Reverse Transcription Kit (Applied Biosystems, cat. no. 4368814). Then the cDNA products were mixed with SYBR green reagent (SsoAdvanced Universal SYBR Green Supermix, Bio‐Rad, cat. no. 1725271). The qPCR analysis was performed by QuantStudio 3 (ThermoFisher).

### Microencapsulation of Engineered Cells

The stable HEK‐293T cells were microencapsulated into coherent alginate‐poly (*L*‐lysine) (PLL)‐alginate beads (400 µm in diameter, 1000–1500 cells per capsule) using an Inotech Encapsulator Research Unit IE‐50R (EncapBioSystems Inc., Greifensee, Switzerland) set to the following parameters: 200 µm nozzle with a vibration frequency of 1025 Hz, a 20 mL syringe operated at a flow rate of 400 units per min and 1200 V bead dispersion voltage.

### Animal Experiments

To establish type‐1 diabetic mice, 8‐week‐old wild‐type male Swiss mice (Janvier Labs) were fasted for 8 h. After fasting, each mouse was injected with freshly diluted STZ (cat. no. S0130, Sigma‐Aldrich) at a dosage of 105 mg kg^−1^/day in 200 µL sodium citrate buffer (adjusted to pH 4.3) for three consecutive days. After one week, fasting blood glucose and insulin levels of the STZ‐treated mice were profiled to confirm the experimental type‐1 diabetes. For glucose‐tolerance tests, the mice were injected with 1.5 g kg^−1^ glucose intraperitoneally and the blood glucose levels were recorded at the indicated time points. Insulin was profiled in blood serum produced using Microtainer serum separator tubes (cat. no. 365967, Becton Dickinson). All experiments involving animals were performed according to the directives of the European Community Council (2010/63/EU), approved by the French Republic (project no. DR2018‐40v5 and APAFIS no. 16753), and performed by Jinbo Huang, Shuai Xue and Ghislaine Charpin‐El Hamnri (no. 69266309) at the University of Lyon, Institut Universitaire de Technologie (IUT), F69622 Villeurbanne, France.

### Glucose

Blood‐glucose levels were profiled using clinically licensed ContourNext test strips and ContourNext ONE reader (Ascensia Diabetes Care).

### SEAP Quantification in Blood Serum

The SEAP levels in the mouse blood were quantified by a luminescence‐based assay kit (ab133077, Abcam, Cambridge, UK).

### Blood Insulin Quantification

Blood insulin levels were profiled by an ELISA kit (cat. no. 10‐1247‐01, Mercordia).

### Animal Blood Sampling

Blood samples were collected from the tail or saphenous veins of mice using a 20 µL glass micro‐haematocrit capillary (Avantor VWR, cat. no. 521–9100), then transferred into blood collection tubes (BD Microtainer, cat. no.: BDAM365968) and centrifuged at 6000× g for 3 min. The supernatant serum was analyzed within 1 h.

### Statistics and Reproducibility

All presented data are representative of two independent experiments unless otherwise stated. The data presentation, sample size of biological replicates (n), statistical analysis, and significance of differences are shown in the figures or given in the corresponding figure legends. Statistical evaluation was performed to examine the significance of differences between two or multiple datasets using the unpaired, two‐tailed Student's *t*‐test or one‐way ANOVA analysis. Microsoft Excel (v16.51, Microsoft Inc.) and GraphPad Prism (v 9.2.0, GraphPad Software Inc.) were used for data processing and analysis.

## Conflict of Interest

The authors declare no conflict of interest.

## Author Contributions

J.H., S.X., A.P.T., and M.F. designed the project. J.H. performed the cell culture experiments. J.H. and S.X. performed animal experiments. J.H., S.X., A.P.T., and M.F. designed the experiments and analyzed the results. J.H., S.X., A.P.T., and M.F. wrote the manuscript.

## Supporting information

Supporting Information

## Data Availability

The data that support the findings of this study are available from the corresponding author upon reasonable request.
