# Supplementary material for: A Gene‐Switch Platform Interfacing with Reactive Oxygen Species Enables Transcription Fine‐Tuning by Soluble and Volatile Pharmacologics and Food Additives
Source: Adv Sci (Weinh). 2024 Mar 25;11(20):2306333. doi: 10.1002/advs.202306333 (PMC11132055; doi:10.1002/advs.202306333)
Supplement: Supplementary file 1 — Supporting Information [file ADVS-11-2306333-s001.pdf]

ADVANCED SCIENCE
Open Access

Supporting Information

for *Adv. Sci.*, DOI 10.1002/advs.202306333

A Gene-Switch Platform Interfacing with Reactive Oxygen Species Enables Transcription Fine-Tuning by Soluble and Volatile Pharmacologics and Food Additives

*Jinbo Huang, Shuai Xue, Ana Palma Teixeira and Martin Fussenegger**

Supplementary Information

A gene switch platform interfacing with reactive oxygen species enables transcription fine-tuning by soluble and volatile pharmacologics and food additives

Jinbo Huang[1], Shuai Xue[1], Ana Palma Teixeira[1], Martin Fussenegger[1,2,*]

[1]Department of Biosystems Science and Engineering, ETH Zurich, Mattenstrasse 26, CH-4058 Basel, Switzerland.

[2]Faculty of Science, University of Basel, Mattenstrasse 26, CH-4058 Basel, Switzerland.

[*]Corresponding author. E-mail: martin.fussenegger@bsse.ethz.ch

This file includes:

- Supplementary Figures: 1-10
- Supplementary Tables: 1-2
- References for supplementary

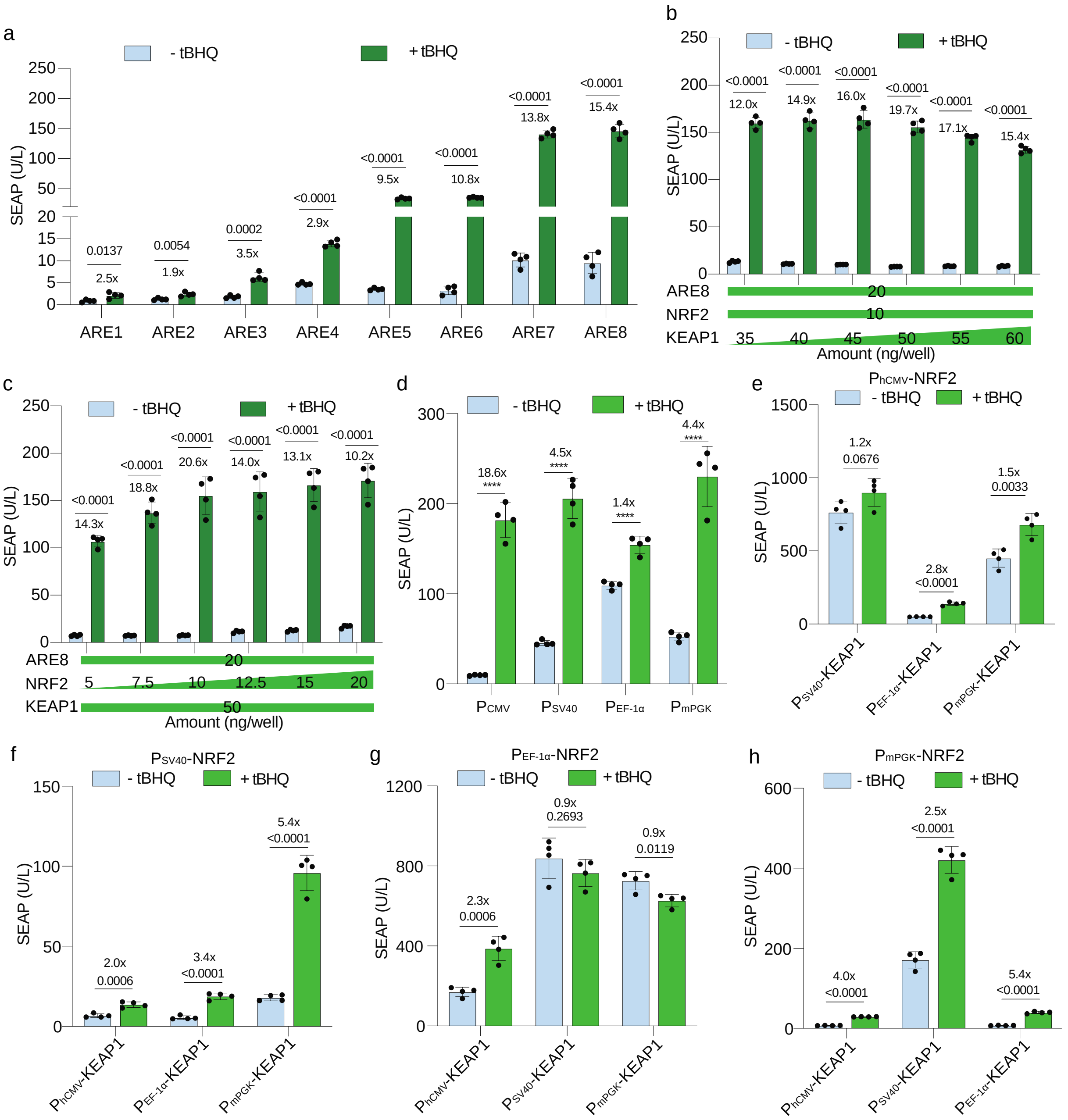

Supplementary Figure 1 | Design and characterization of the ROS$_{\text{SENSE}}$ system. a. Performance comparison of ROS$_{\text{SENSE}}$-engineered cells containing ARE variants having one repeat to eight repeats. **b**. The ROS$_{\text{SENSE}}$ system was transiently transfected with constant amounts of pJH1227 (P$_{\text{ARE8}}$-SEAP-pA) and pJH1003 (P$_{\text{hCMV}}$-NRF2-pA). The indicated amount of pJH1004 (P$_{\text{hCMV}}$-KEAP1-pA) was applied per well of a 96-well plate cell culture. **c.** The ROS$_{\text{SENSE}}$ system was transiently transfected with constant amounts of pJH1227 (P$_{\text{ARE8}}$-SEAP-pA) and pJH1004 (P$_{\text{hCMV}}$-KEAP1-pA). The indicated amount of pJH1003 (P$_{\text{hCMV}}$-NRF2-pA) was applied per well of a 96-well plate cell culture. **d-h**. Performance comparison of the ROS$_{\text{SENSE}}$ system transfected with NRF2 and KEAP1 combinations with different promoters. **d.** Promoter screening for NRF2 and KEAP1. Both NRF2 and KEAP1 were

constitutively expressed with the indicated promoters. P_{hCMV}: human cytomegalovirus immediate early promoter; P_{SV40}: simian virus 40 promoter; $P_{EF\text{-}1\alpha}$, human elongation factor-1 alpha promoter; P_{mPGK}, murine phosphoglycerate kinase promoter. **e**. P_{hCMV}-controlled NRF2 with KEAP1 containing distinct promoters; **f**. P_{SV40}-controlled NRF2 with KEAP1 containing the indicated promoters; **g**. $P_{EF\text{-}1\alpha}$-controlled NRF2 with KEAP1 containing the indicated promoters; **h**. P_{mPGK}-controlled NRF2 with KEAP1 containing the indicated promoters. tBHQ (40 µmol) was added in the treatment group (+ tBHQ), and the non-treated groups received an equivalent amount of solution (- tBHQ). Data points represent mean ± SD; n = 4. The *P* values above the bars indicate the significance of differences in the mean values (versus the non-induced or indicated group). The fold increase is also shown.

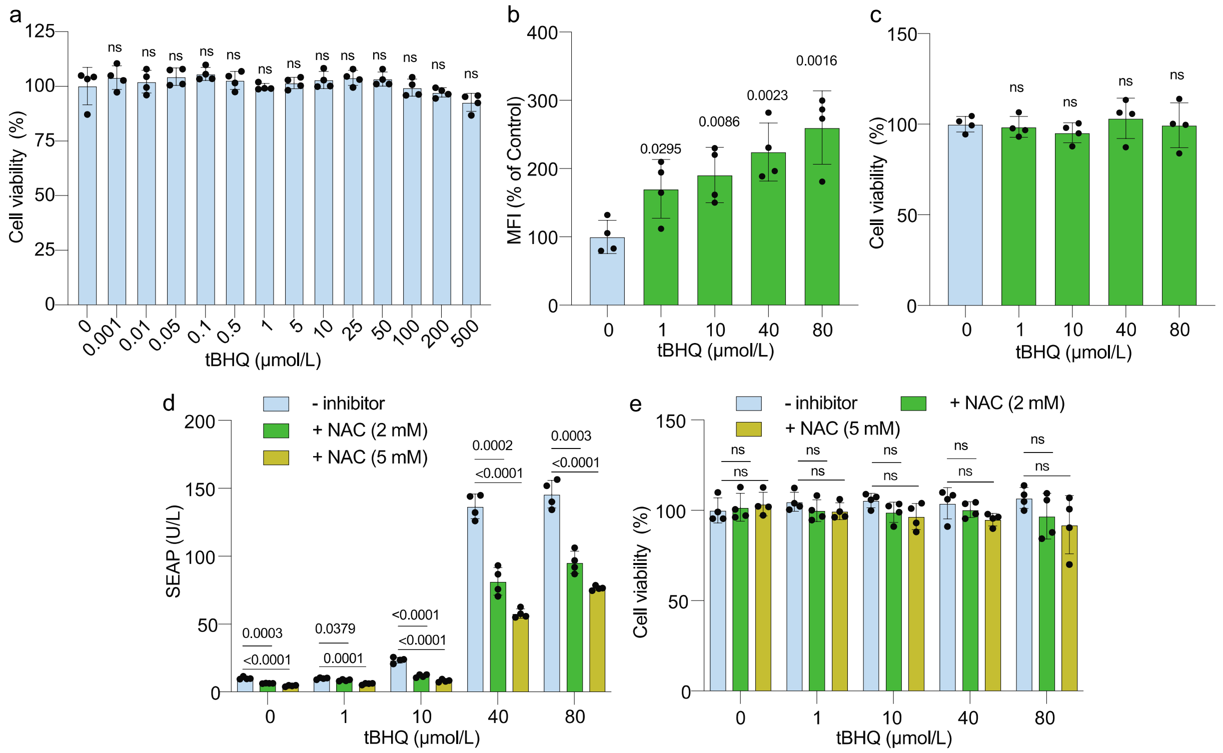

Supplementary Figure 2 | Validation of the **ROS$_{SENSE}$ system. a.** Viability of ROS$_{SENSE}$-transfected HEK-293T cells was measured after treatment with tBHQ at the indicated concentrations. Cell viability was assayed using resazurin assay. **b**. Quantitative assay of ROS induced by tert-butylhydroquinone (tBHQ) at the indicated concentrations. **c**. Cell viability was analyzed after treating the cells with the indicated concentration of tBHQ. **d**. Effect of ROS inhibitor on ROS$_{SENSE}$-engineered HEK-293T cells. NAC: N-acetyl-l-cysteine. The cells were first incubated with the indicated concentration of NAC for 30 min, then the indicated

concentrations of tBHQ were added. **e**. Cell viability was analyzed 24 hours after the addition of NAC and tBHQ for the cells from (**d**). SEAP production was analyzed after treatment with tBHQ for 24 h. MFI: mean fluorescence intensity. ns means not significant (*P* value > 0.05). Data points represent mean ± SD; n = 4. The *P* values above the bars indicate the significance of differences in the mean values (versus the non-induced group or the indicated group).

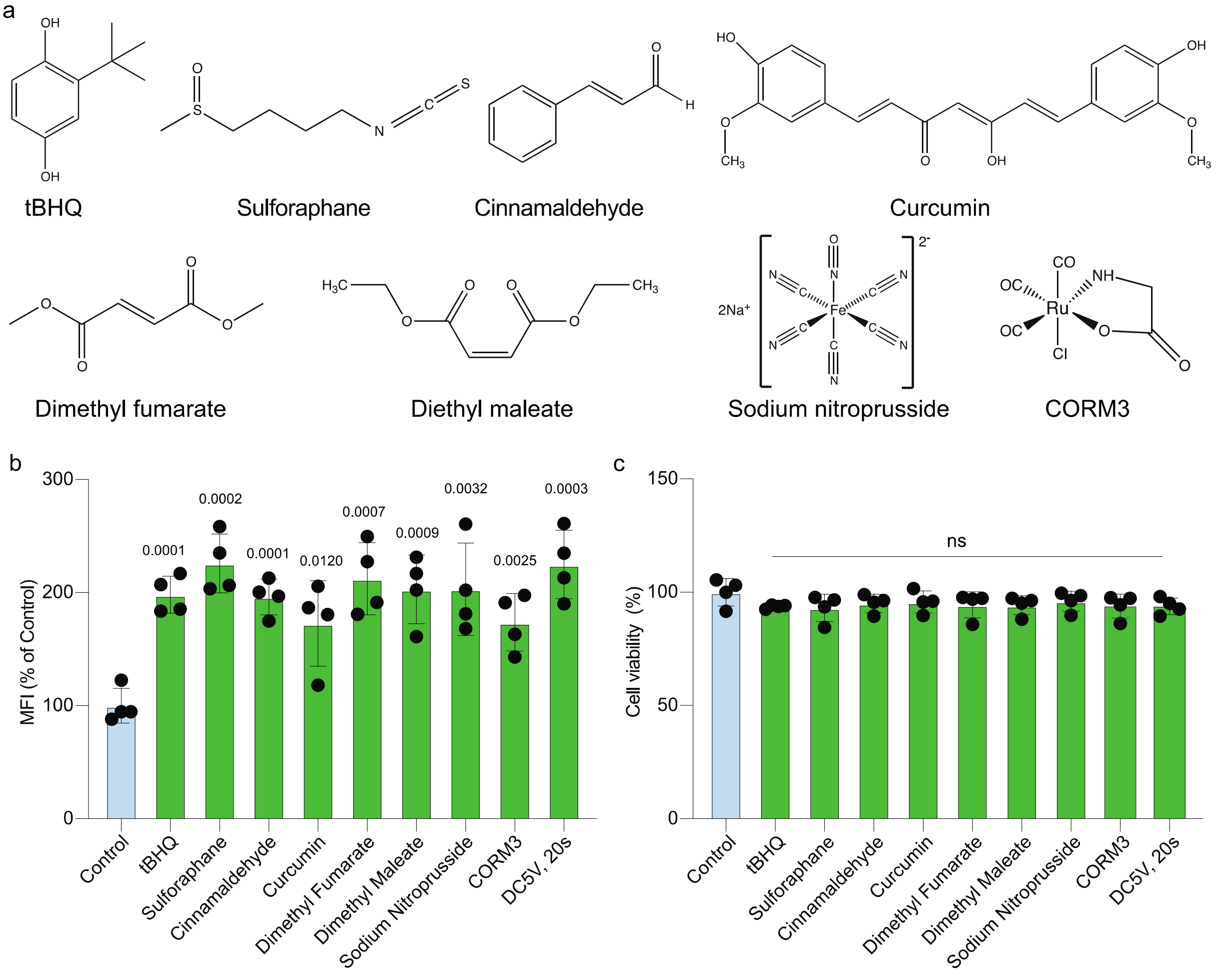

Supplementary Figure 3 | Profiling ROS levels and viability of cells treated with various chemicals and cells in the direct current (DC)-stimulated group. a. Molecular structures of the ROS-enhancing molecules used in this study. **b**. Quantitative assay of ROS induced by the indicated chemical compounds. **c**. Cell viability was analyzed after treating the cells with the indicated chemicals. MFI: mean fluorescence intensity. The concentrations of sulforaphane, cinnamaldehyde, curcumin, dimethyl fumarate, diethyl maleate, sodium nitroprusside, CORM3 and tBHQ were 10, 50, 50, 100, 100, 400, 500 and 40 μmol/L, respectively. In the case of electrostimulated cells, direct current was applied for 20 s (DC 5 V, 20 s). Data points represent mean ± SD; n = 4. The *P* values above the bars indicate the significance of differences

in the mean values (versus the control group). ns means not significant (*P* value > 0.05, versus the control group).

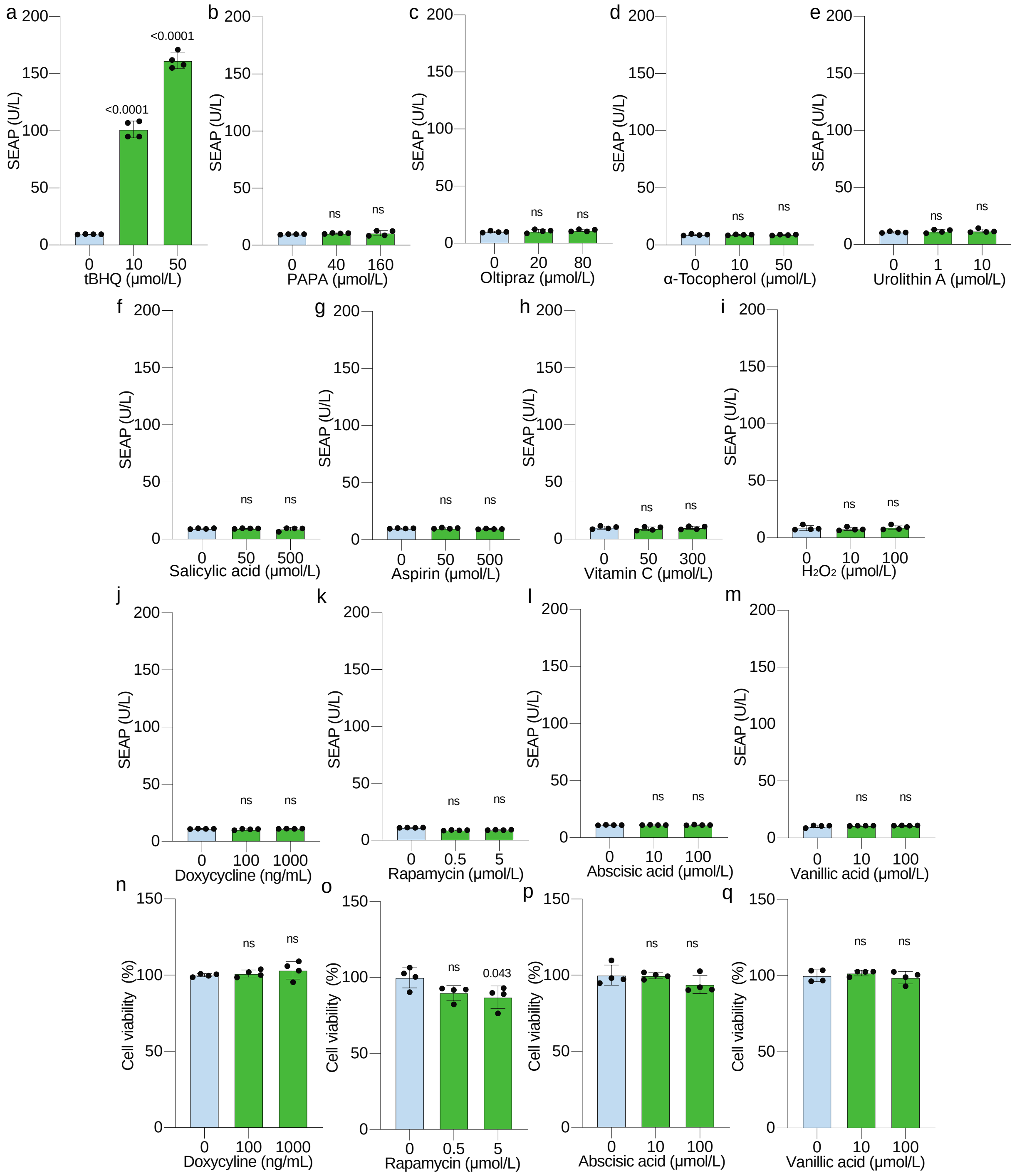

Supplementary Figure 4 | Effects of additional chemicals on ROS_{SENSE}-engineered HEK-293T cells. a-i. SEAP production was analyzed after treating the cells with tBHQ (**a**), PAPA

(PAPA NONOate) (**b**), oltipraz (**c**), α-tocopherol (**d**), urolithin A (**e**), salicylic acid (**f**), aspirin (acetylsalicylic acid) (**g**), vitamin C (**h**), or H_2O_2 (**i**), at the indicated concentrations. **j-m.** SEAP production was analyzed after treating the cells with conventional small molecules used as gene switch inducers: doxycycline (**j**), rapamycin (**k**), abscisic acid (**l**), vanillic acid (**m**). **n-q,** Cell viability measurement for conventional molecule-induced cells from **j-m**, respectively. Cell viability was determined by resazurin assay. Data points represent mean ± SD, n = 4. ns means not significant (*P* value > 0.05). The *P* values above the bars indicate the significance of differences in the mean values (versus the non-induced group).

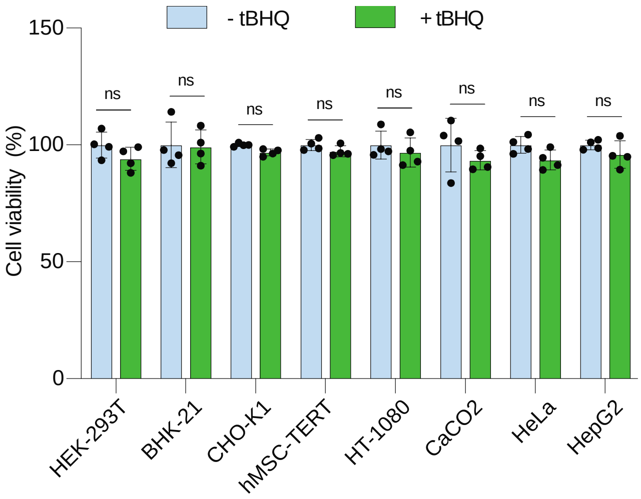

Supplementary Figure 5 | Viability of ROS$_{SENSE}$-transfected mammalian cells treated with tBHQ. Viability of ROS$_{SENSE}$-transfected mammalian cells without (- tBHQ) or with (+tBHQ) tBHQ treatment (40 μmol/L). Cell viability was assayed using resazurin assay. Data points represent mean ± SD; n = 4. ns means not significant (*P* value > 0.05 versus the corresponding untreated group).

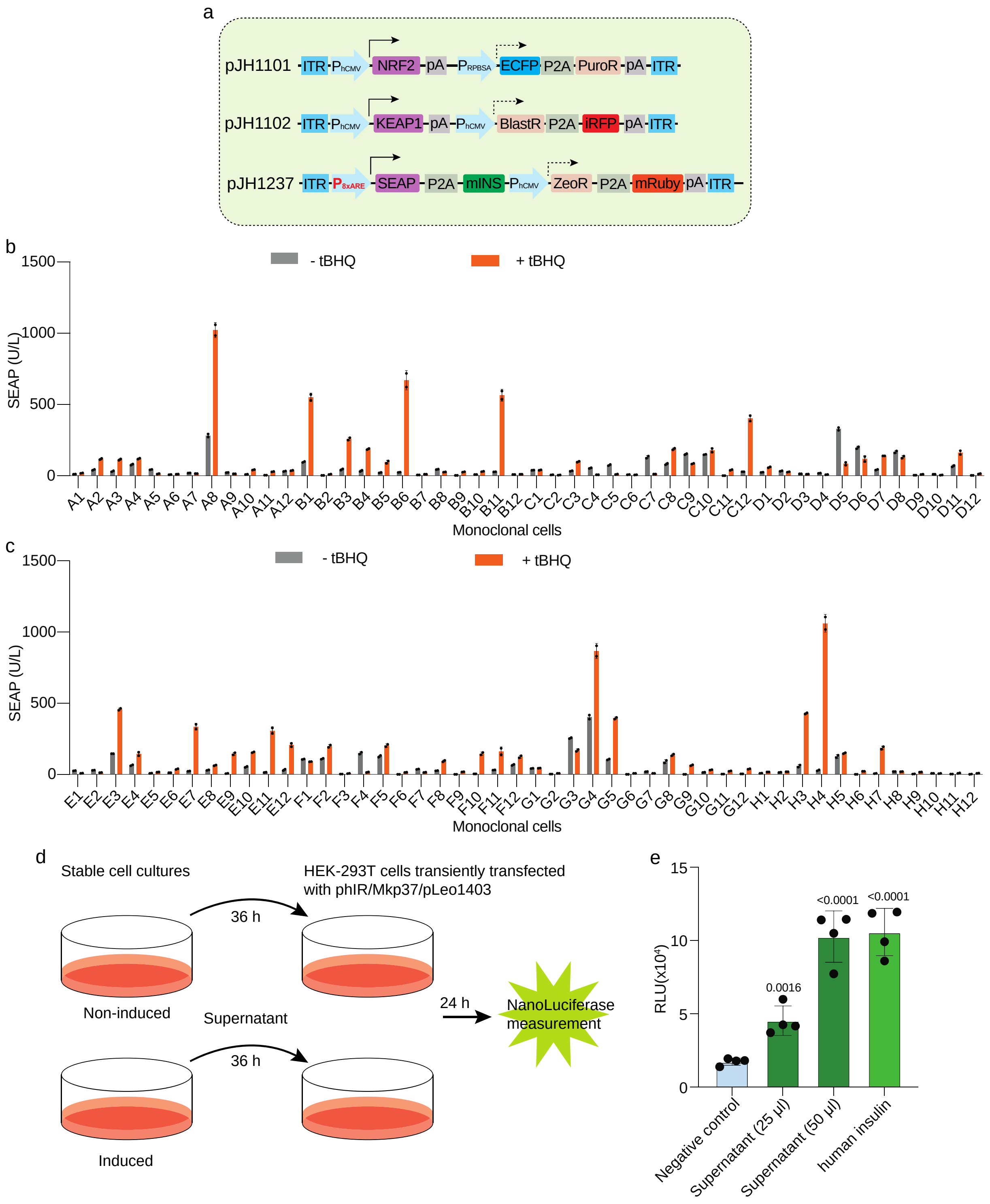

Supplementary Figure 6 | Generation and screening of monoclonal cell lines. a. Construction design of plasmids used for the generation of monoclonal cell lines. The stable cell line is based on the constitutive expression of NRF2 (ITR-P$_{\text{hCMV}}$-NRF2-pA:P$_{\text{RPBSA}}$-ECFP-P2A-PuroR-pA-ITR, pJH1101), KEAP1 (ITR-P$_{\text{hCMV}}$-KEAP1-pA:P$_{\text{hCMV}}$-BlastR-P2A-iRFP-pA-ITR, pJH1102) and eight-tandem ARE-controlled SEAP followed by mouse insulin (mINS) (ITR-P$_{\text{ARE8}}$-SEAP-P2A-mINS-pA: P$_{\text{hCMV}}$-ZeoR-P2A-mRuby-pA-ITR, pJH1237). All the

constructs contain flanking inverted terminal repeats (ITR) for the recognition of SB100X transposase. **b,c**. Screening of monoclonal HEK-293T cell line for plate 1 (**b**) and plate 2 (**c**). HEK-293T cells were stably transfected with pJH1101 (ITR-P_{hCMV}-NRF2-pA:P_{RPBSA}-ECFP-P2A-PuroR-pA-ITR), pJH1102 (ITR-P_{hCMV}-KEAP1-pA:P_{hCMV}-BlastR-P2A-iRFP-pA-ITR) and pJH1237 (ITR-P_{ARE8}-SEAP-P2A-mINS-pA:P_{hCMV}-ZeoR-P2A-mRuby-pA-ITR). Two 48-well plates of monoclonal cell lines were randomly selected by profiling SEAP production after induction with tBHQ (40 μmol/L) for 24 h. **d**. Scheme of insulin-activity bioassay. ROS_{SENSE}-INS stable cells were cultured in inducer-containing or inducer-free medium for 36 h. Then the supernatant was transferred to HEK-293T cells transiently transfected with phIR (P_{hCMV}-hIR-pA), Mkp37 (P_{hCMV}-TetR-Elk1-pA) and pLeo1403 (P_{TRE}-NanoLuc-pA). Nano-luciferase (NanoLuc) quantification was performed after 24 h. **e**. NanoLuc levels in insulin-activity bioassay as a readout of insulin activity. Human insulin (1 μg/L) was used as a positive control. The SEAP or NanoLuc levels in the culture supernatant were quantified after induction for 24 h. RLU: relative luciferase units. Abbreviations for constructs are listed after **Supplementary Table 1**. Data points represent mean ± SD; in (**b**) and (**c**), n = 2; in (**e**), n = 4. The *P* values above the bars indicate the significance of differences in the mean values (versus the negative control group).

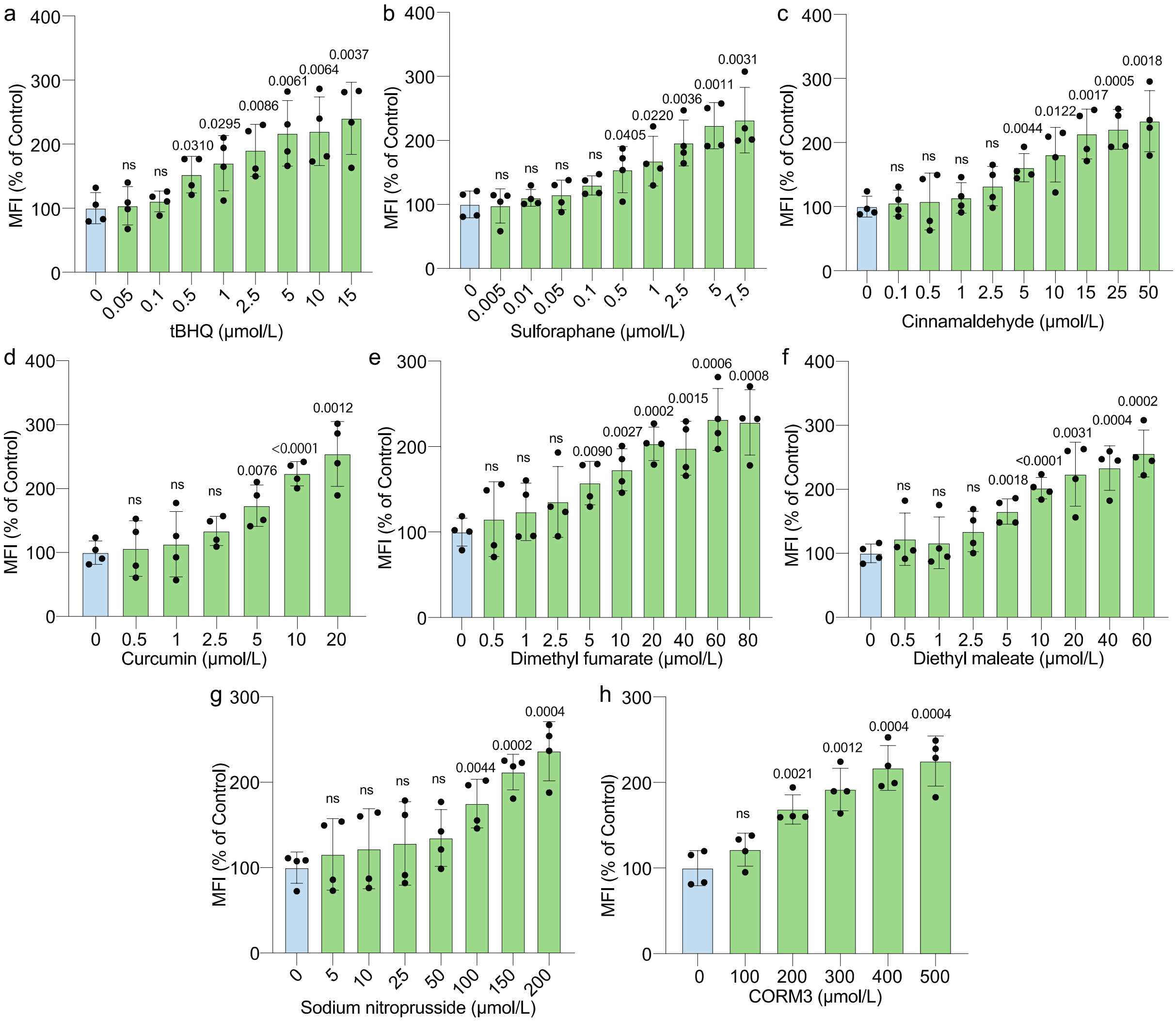

Supplementary Figure 7 | Quantification of ROS in ROS$_{SENSE}$-INS cells induced by various chemicals. a-h. ROS generation was analyzed after treating ROS$_{SENSE}$-INS cells with tert-butylhydroquinone (tBHQ) (**a**), sulforaphane (**b**), cinnamaldehyde (**c**), curcumin (**d**), diethyl maleate (**e**), dimethyl fumarate (**f**), sodium nitroprusside (**g**) or CORM3 (**h**) at the indicated concentrations. MFI: mean fluorescence intensity. Data points represent mean ± SD, n = 4. ns means not significant (*P* value > 0.05). The *P* values above the bars indicate the significance of differences in the mean values (versus the corresponding non-induced group).

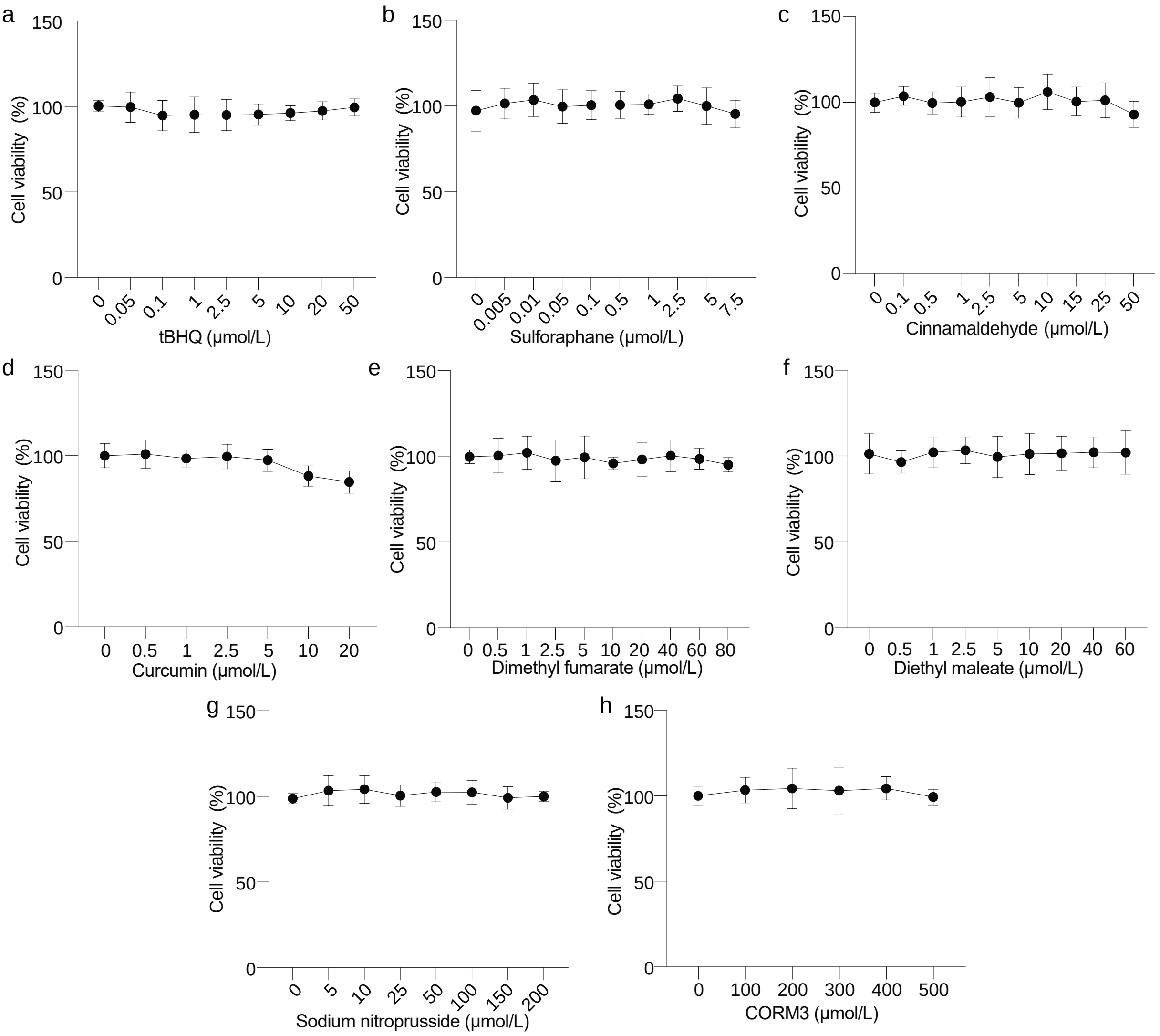

Supplementary Figure 8 | Effects of various inducing chemicals on ROS$_{SENSE}$-INS cells. a-h. Cell viability was analyzed after treating the ROS$_{SENSE}$-INS cells with tert-butylhydroquinone (tBHQ) (**a**), sulforaphane (**b**), cinnamaldehyde (**c**), curcumin (**d**), dimethyl fumarate (**e**), diethyl maleate (**f**), sodium nitroprusside (**g**), or CORM3 (**h**) at the indicated concentrations. Data points represent mean ± SD, n = 4.

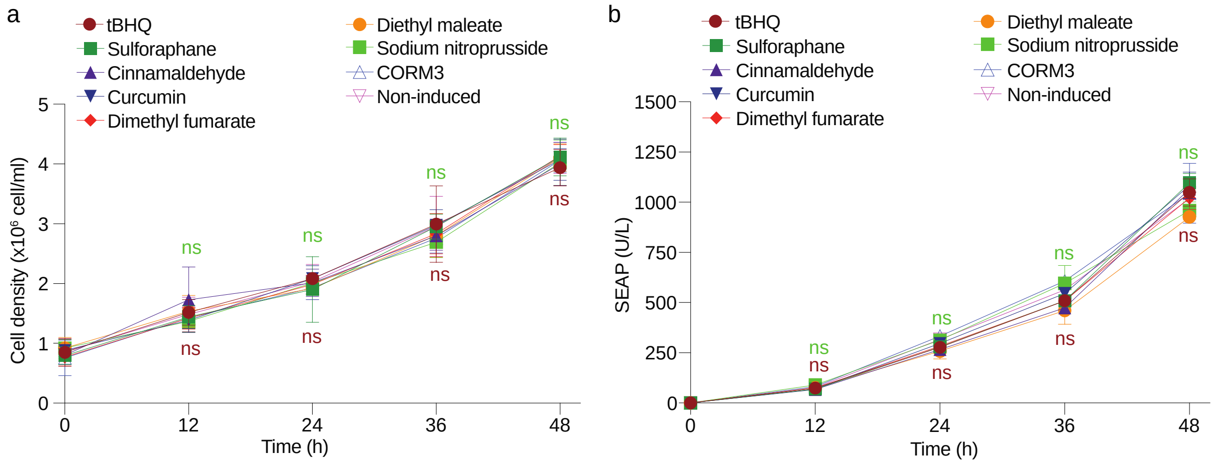

Supplementary Figure 9 | Impact of chemicals on cell growth and recombinant protein production. a,**b**, Time courses of cell density (**a**) and recombinant SEAP expression (**b**) after treatment with the indicated chemicals at the EC50 concentrations shown in **Fig. 3**. Wild-type HEK-293T cells with constitutive expression of SEAP (transfected with pJH3, P_{hCMV}-SEAP-pA) were treated with chemicals and analyzed every 12 h during 48 h of culture. Data points represent mean ± SD, n = 4. ns means not significant. The colored statistical marks refer to the corresponding data points with the same color.

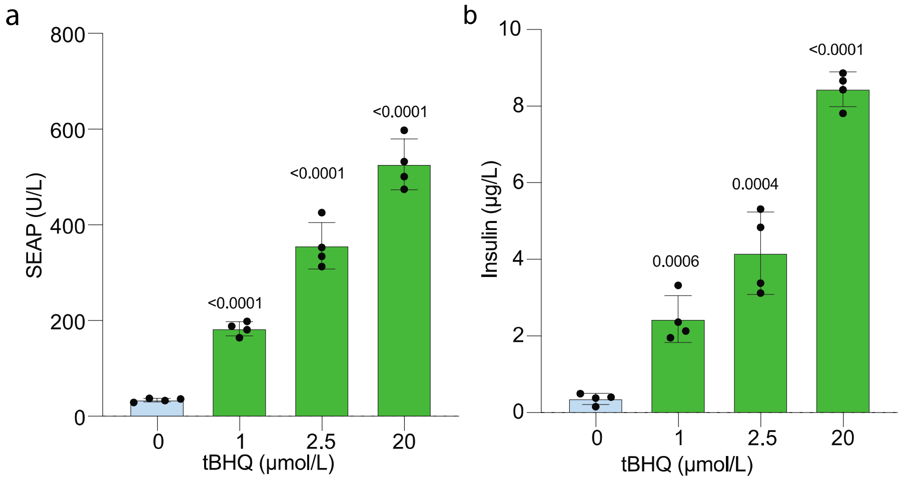

Supplementary Figure 10 | SEAP and insulin production by alginate-encapsulated ROS_{SENSE}-INS cells. a,b. The microencapsulated cells were cultured in 24-well plates with DMEM medium containing 10% fetal bovine serum, and induced with tBHQ at the indicated concentrations. The SEAP (**a**) and insulin (**b**) levels were quantified in the cell culture

supernatant after induction for 24 h. Columns are each the mean ± SD of the four determinations indicated by filled circles. The *P* values above the bars indicate the significance of differences in the mean values (versus the non-induced group).

Supplementary Table 1. Plasmids used and designed in this study

**Plasmid**	**Description**	**Reference**
BB6-BlastR	SB100X-specific transposon containing a constitutive BlastR and iRFP expression unit (ITR-MCS:P_{hCMV}-BlastR-P2A-iRFP-pA-ITR).	Huang et al. 2023
BB6-PuroR	SB100X-specific transposon containing a constitutive ECFP and PuroR expression unit (ITR-MCS:P_{RPBSA}-ECFP-P2A-PuroR-pA-ITR).	Huang et al. 2023
BB6-ZeoR	SB100X-specific transposon containing a constitutive ZeoR and mRuby expression unit (ITR-MCS-pA: P_{hCMV}-ZeoR-P2A-mRuby-pA-ITR).	Huang et al. 2023
H107	Constitutive mammalian expression vector. (P_{hCMV}-eGFP-3FLAG:P_{mPGK}-ZeoR-pA).	ObiO, Shanghai
Mkp37	Constitutive mammalian TetR-Elk1 fusion protein expression vector (P_{hCMV}-TetR-Elk1-pA).	Keeley et al.[1]
pcDNA3.1(+)	Constitutive mammalian expression vector containing a NeoR resistance gene (P_{hCMV}-MCS-pA).	Thermo Fisher Scientific, CA
pCMV-T7-SB100	Constitutive SB100X expression vector (P_{hCMV}-SB100X-pA) (Addgene no. 34879).	Mates et al.[2, 3]
pCK53	CRE-driven SEAP expression vector (P_{CRE}-SEAP-pA).	Kemmer et al.[4]

phIR	Constitutive mammalian expression vector containing human insulin receptor (hIR) gene (P_{hCMV}-hIR-pA).	Ye et al. [5]
pREP-8xARE-GFP	8xARE-driven GFP expression in pMIR vector (P_{8xARE}-GFP-pA) (Addgene no. 134910)	Wyler et al.[6]
pKeap1-XL	*Keap1* from *Xenopus laevis* in pDONR223 vector (DNASU no. XICD00711457).	DNASU, USA
pNrf2-XL	*Nrf2* from *Xenopus laevis* in pDONR223 vector (DNASU no. XICD00716052).	DNASU, USA
pdCas9-VPR	Constitutive dCas9-VPR expression vector (P_{hCMV}- dCas9-VPR-pA) (Addgene no. 63798).	Chavez et al.[7]
pKEAP1	Constitutive KEAP1 expression vector (P_{hCMV}-KEAP1-3xFlag-pA) (Addgene no. 28023).	Fan et al.[8]
pLeo1403	Mammalian reporter plasmid for TetR-Elk1-induced NanoLuc expression (P_{TRE}-NanoLuc-pA).	Maysam et al.[9]
pMF111	Mammalian reporter plasmid for TetR-Elk1-induced SEAP expression (P_{TRE}-SEAP-pA).	Fussenegger et al.[10]
pMX256	SB100X-specific transposon containing a P_{NFAT5}-driven SEAP and mouse insulin expression unit and a constitutive eGFP and ZeoR expression unit (ITR-P_{NFAT5}-SEAP-P2A-mINS-pA:P_{RPBSA}-eGFP-P2A-ZeoR-pA-ITR).	Xie et al.[11]
pNRF2	Constitutive NRF2 expression vector (P_{hCMV}-Flag-NRF2-pA) (Addgene no. 36971).	Camp et al.[12]
pXS101	SB100X-specific transposon containing a constitutive mammalian promoter-driven CREB1-TetR stable expression vector in mammalian cells (ITR-P_{hCMV}-CREB1-TetR-2A-mCherry-2A-PuroR-pA-ITR).	Xue et al.[13]
pJH3	Constitutive mammalian SEAP expression vector (P_{hCMV}-SEAP-pA).	Huang et al. 2023
pJH42	Constitutive SB100X expression vector (P_{hCMV}-SB100X-pA).	Huang et al. 2023

pJH43	Constitutive mammalian expression vector containing a NeoR resistance gene (P_{SV40}-MCS-pA).	Huang et al. unpublished
pJH44	Constitutive mammalian expression vector containing a NeoR resistance gene ($P_{EF\text{-}1\alpha}$-MCS-pA).	Huang et al. unpublished
pJH45	Constitutive mammalian expression vector containing a NeoR resistance gene (P_{mPGK}-MCS-pA).	Huang et al. unpublished
pJH1003	Constitutive NRF2 expression vector (P_{hCMV}-NRF2-pA).	Huang et al. 2023
pJH1004	Constitutive KEAP1 expression vector (P_{hCMV}-KEAP1-pA).	Huang et al. 2023
pJH1005	DART-driven SEAP expression vector (P_{DART1}-SEAP-pA).	Huang et al. 2023
pJH1006	Two tandem DART-driven SEAP expression vector (P_{DART2}-SEAP-pA).	Huang et al. 2023
pJH1009	Three tandem DART-driven SEAP expression vector (P_{DART3}-SEAP-pA).	Huang et al. 2023
pJH1010	Four tandem DART-driven SEAP expression vector (P_{DART4}-SEAP-pA).	Huang et al. 2023
pJH1040	SEAP-2A-mINS expression driven by a synthetic DART promoter containing antioxidative response elements (ARE) (P_{DART}-SEAP-2A-mINS-pA).	Teixeira et al., 2023
pJH1053	SB100X-specific transposon containing a constitutive NRF2 and ZeoR expression unit (ITR-P_{hCMV}-NRF2-P2A-ZeoR-pA-ITR).	Huang et al. 2023
pJH1054	SB100X-specific transposon containing a constitutive KEAP1 and BlastR expression unit (ITR-P_{hCMV}-KEAP1-P2A-BlastR-pA-ITR).	Huang et al. 2023

pJH1089	ARE-driven NanoLuc expression vector (P_{DART}-NanoLuc-pA).	Huang et al. 2023
pJH1090	ARE-driven NanoLuc and mouse insulin expression vector (P_{DART}-NanoLuc-P2A-mINS-pA).	Huang et al. 2023
pJH1096	SB100X-specific transposon ARE-driven NanoLuc and mouse insulin expression unit, and a constitutive ZeoR expression unit<br>(ITR-P_{DART}-NanoLuc-P2A-mINS: mPGK-ZeoR-pA-ITR)	Huang et al. 2023
pJH1101	SB100X-specific transposon containing a constitutive NRF2 expression unit and a constitutive ECFP and PuroR expression unit<br>(ITR-P_{hCMV}-NRF2-pA: P_{RPBSA}-ECFP-P2A-PuroR-pA-ITR).	Huang et al. 2023
pJH1102	SB100X-specific transposon containing a constitutive KEAP1 expression unit and a constitutive BlastR and iRFP expression unit<br>(ITR-P_{hCMV}-KEAP1-pA:P_{hCMV}-BlastR-P2A-iRFP-pA-ITR).	Huang et al. 2023
pJH1157	Four tandem ARE-driven SEAP and mouse insulin expression vector<br>(P_{DART4}-SEAP-P2A-mINS-pA)	Huang et al. 2023
pJH1159	SB100X-specific transposon containing a four tandem ARE-driven SEAP and insulin expression unit and a constitutive ECFP and PuroR expression unit<br>(ITR-P_{DART4}-SEAP-P2A-mINS-pA:P_{RPBSA}-ECFP-P2A-PuroR-pA-ITR).	Huang et al. 2023
pJH1169	SB100X-specific transposon containing a four tandem ARE-driven SEAP and insulin expression unit and a constitutive ZeoR expression unit<br>(ITR-P_{DART4}-SEAP-P2A-mINS:P_{mPGK}-ZeoR-pA-ITR).	Huang et al. 2023

pJH1175	Constitutive NRF2 fused with VP64 vector (P_{hCMV}-NRF2-VP64-pA).	Huang et al. 2023
pJH1181	Constitutive NRF2 fused with TetR and VP64 vector (P_{hCMV}-NRF2-TetR-VP64-pA).	Huang et al. 2023
pJH1214	DART-driven short-lived eGFP expression vector (P_{DART}-slGFP-pA).	Huang et al. 2023
pJH1027	DART-driven GFP expression vector (P_{DART}-GFP-pA).<br>The fragment was PCR-amplified from H107 with OJH1027-GF (5'-CGAAGCGGAATTCACCATGACTAGTGTGAGCAAGGGCGAGGAGC-3') and OJH1027-GR (5'-AGCTTTCTAGACACCGGTGGATCCCTACTTGTACAGCTCGTCCATGC-3'), and cloned into pJH1005 (digested by *Spe*I/*Bam*HI) by Gibson assembly[14].	This work
pJH1207	Constitutive Nrf2-XL expression vector (P_{hCMV}-Nrf2-XL-pA).<br>The fragment was PCR-amplified from pNrf2-XL with OJH1207-GF (5'-AAGCTGTTCGAAGCGGAATTCACCATGATGGAGATCGAGATACCCCTTC-3') and OJH1208-GR (5'-AAGCTTTCTAGACACCGGTGGATCCCTATTCCTTCTTTATGCTAACTTTCTTGGTTTTTG-3'), and cloned into pJH3 (digested by *Eco*RI/*Bam*HI) by Gibson assembly[14].	This work
pJH1208	Constitutive Keap1-XL expression vector (P_{hCMV}-Keap1-XL-pA).<br>The fragment was PCR-amplified from pKeap1-XL with OJH1208-GF (5'-AAGCTGTTCGAAGCGGAATTCACCATGTACGCGACGGAGTGCC-3') and OJH1208-GR (5'-GCTTTCTAGACACCGGTGGATCCCTATTTTTGGTGT	This work

	GTGAGGAGAAGCAAC-3'), and cloned into pJH3 (digested by *Eco*RI/*Bam*HI) by Gibson assembly.	
pJH1219	Eight tandem ARE-driven SEAP expression in pMIR vector (P_{ARE8}-SEAP-pA)<br>The fragment was PCR-amplified from pJH1005 with OJH1208-GF (5'-GGTACTGTTGGTAAACTCGAGGAATTCACCATGACTAGTCTGCTGC-3') and OJH1208-GR (5'-CGGCAGATTCGAGCAGACCGGATCCACCGGTGTCTAGAAAGCTTCATATGGTGCACTCTCAGTA-3'), and cloned into pREP-8xARE-GFP (Addgene no. 134910) (digested by *Eco*RI/*Bam*HI) by Gibson assembly.	This work
pJH1220	ARE-driven SEAP expression in pcDNA3.1 vector (P_{ARE1}-SEAP-pA).<br>The fragment was PCR-amplified from pJH1219 with OJH1220-GF (5'-GATCGGGAGATCTCCACGCGTGTGCAGGTGCGTGACACAGCAAGACACTAGAGGGT-3') and OJH1220-1227-GR (5'-TGCAGGCCGGCCTCAAAGCTTTCTAGACACCGGTGGATCC-3'), and cloned into pJH3 (digested by *Mlu*I/*Bam*HI) by Gibson assembly.	This work
pJH1221	Two tandem ARE-driven SEAP expression in pcDNA3.1 vector (P_{ARE2}-SEAP-pA).<br>The fragment was PCR-amplified from pJH1220 with OJH1221-GF (5'-GGATCGGGAGATCTCCACGCGTGTGCAGGTGCGTGACGTAGCATCGGTGACACAGC-3') and OJH1220-1227-GR (5'-TGCAGGCCGGCCTCAAAGCTTTCTAGACACCGGTGGATCC-3'), and cloned into pJH3 (digested by *Mlu*I/*Bam*HI) by Gibson assembly.	This work

pJH1222	Three tandem ARE-driven SEAP expression in pcDNA3.1 vector (P_{ARE3}-SEAP-pA).<br><br>The fragment was PCR-amplified from pJH1221 with OJH1222-GF (5'-GGATCGGGAGATCTCCACGCGTGTGCAGGTGCGTGACTAGGCAAGCGTGACGTAG-3') and OJH1220-1227-GR (5'-TGCAGGCCGGCCTCAAAGCTTTCTAGACACCGGTGGATCC-3'), and cloned into pJH3 (digested by *Mlu*I/*Bam*HI) by Gibson assembly.	This work
pJH1223	Four tandem ARE-driven SEAP expression in pcDNA3.1 vector (P_{ARE4}-SEAP-pA).<br><br>The fragment was PCR-amplified from pJH1222 with OJH1223-GF (5'-GGATCGGGAGATCTCCACGCGTGTGCAGGTGCGTGACAAAGCACCCGTGACTAGGC-3') and OJH1220-1227-GR (5'-TGCAGGCCGGCCTCAAAGCTTTCTAGACACCGGTGGATCC-3'), and cloned into pJH3 (digested by *Mlu*I/*Bam*HI) by Gibson assembly.	This work
pJH1224	Five tandem ARE-driven SEAP expression in pcDNA3.1 vector (P_{ARE5}-SEAP-pA).<br><br>The fragment was PCR-amplified from pJH1223 with OJH1224-GF (5'-GGATCGGGAGATCTCCACGCGTGTGCAGGTGCGTGACACTGCACTGGTGACAAAGC-3') and OJH1220-1227-GR (5'-TGCAGGCCGGCCTCAAAGCTTTCTAGACACCGGTGGATCC-3'), and cloned into pJH3 (digested by *Mlu*I/*Bam*HI) by Gibson assembly.	This work

pJH1225	Six tandem ARE-driven SEAP expression in pcDNA3.1 vector (P_{ARE6}-SEAP-pA).<br>The fragment was PCR-amplified from pJH1224 with OJH1225-GF (5'-GGATCGGGAGATCTCCACGCGTGTGCAGGTGCGTGACGATGCAGACGTGACACTGC-3') and OJH1220-1227-GR (5'-TGCAGGCCGGCCTCAAAGCTTTCTAGACACCGGTGGATCC-3'), and cloned into pJH3 (digested by *Mlu*I/*Bam*HI) by Gibson assembly.	This work
pJH1226	Seven tandem ARE-driven SEAP expression in pcDNA3.1 vector (P_{ARE7}-SEAP-pA).<br>The fragment was PCR-amplified from pJH1225 with OJH1226-GF (5'-GGATCGGGAGATCTCCACGCGTGTGCAGGTGCGTGACTACGCATGCGTGACGATGC-3') and OJH1220-1227-GR (5'-TGCAGGCCGGCCTCAAAGCTTTCTAGACACCGGTGGATCC-3'), and cloned into pJH3 (digested by *Mlu*I/*Bam*HI) by Gibson assembly.	This work
pJH1227	Eight tandem ARE-driven SEAP expression in pcDNA3.1 vector (P_{ARE8}-SEAP-pA).<br>The fragment was PCR-amplified from pJH1219 with OJH1227-GF (5'-GGATCGGGAGATCTCCACGCGTGTGCAGGTGCCAGAACATTTCTC-3') and OJH1220-1227-GR (5'-TGCAGGCCGGCCTCAAAGCTTTCTAGACACCGGTGGATCC-3'), and cloned into pJH3 (digested by *Mlu*I/*Bam*HI) by Gibson assembly.	This work
pJH1228	Constitutive human NRF2 expression vector (P_{SV40}-hNRF2-pA).	This work

	The fragment was PCR-amplified from pJH1003 with OJH1228-GF (5'-CAAGCTGTTCGAAGCGGAATTCACCATGACTAGTATGATGGACTTGGAGCTGCC-3') and OJH1228-GR (5'-CAAAGCTTTCTAGACACCGGTGGATCCGTTTTTCTTAACATCTGGCTTCTTACTTTTG-3'), and cloned into pJH43 (digested by *Eco*RI/*Bam*HI) by Gibson assembly.	
pJH1229	Constitutive human NRF2 expression vector ($P_{EF\text{-}1\alpha}$-hNRF2-pA).<br><br>The fragment was PCR-amplified from pJH1003 with OJH1229-GF (5'-GTGAGAGTGTTCGAAGCGGAATTCACCATGACTAGTATGATGGACTTGGAGCTGCC-3') and OJH1229-GR (5'-TTTCTAGACACCGGTGGATCCCTAGTTTTTCTTAACATCTGGCTTCTTAC-3'), and cloned into pJH44 (digested by *Eco*RI/*Bam*HI) by Gibson assembly.	This work
pJH1230	Constitutive human NRF2 expression vector (P_{mPGK}-hNRF2-pA).<br><br>The fragment was PCR-amplified from pJH1003 with OJH1230-GF (5'-CGGTATAGGTTCGAAGCGGAATTCACCATGACTAGTATGATGGACTTGGAG -3') and OJH1230-GR (5'-AAAGCTTTCTAGACACCGGTGGATCCCTAGTTTTTCTTAACATCTGGCTT-3'), and cloned into pJH45 (digested by *Eco*RI/*Bam*HI) by Gibson assembly.	This work
pJH1231	Constitutive human KEAP1 expression vector (P_{SV40}-hKEAP1-pA).<br><br>The fragment was PCR-amplified from pJH1004 with OJH1231-GF (5'-CCAAGCTGTTCGAAGCGGAATTCACCATGACTAGTATGCAGCCAGATCCCAGGC-3') and OJH1231-GR (5'-	This work

	CAAAGCTTTCTAGACACCGGTGGATCCACAGGTACAGTTCTGCTGGTCAATC-3’), and cloned into pJH43 (digested by *Eco*RI/*Bam*HI) by Gibson assembly.	
pJH1232	Constitutive human KEAP1 expression vector ($P_{EF\text{-}1\alpha}$-KEAP1-pA).<br>The fragment was PCR-amplified from pJH1004 with OJH1232-GF (5’-AGAGTGTTCGAAGCGGAATTCACCATGACTAGTATGCAGCCAGATCC-3’) and OJH1232-GR (5’-AAGCTTTCTAGACACCGGTGGATCCACAGGTACAGTTCTGCTGGTC-3’), and cloned into pJH44 (digested by *Eco*RI/*Bam*HI) by Gibson assembly.	This work
pJH1233	Constitutive human KEAP1 expression vector (P_{mPGK}-hKEAP1-pA).<br>The fragment was PCR-amplified from pJH1004 with OJH1233-GF (5’-CGGTATAGGTTCGAAGCGGAATTCACCATGACTAGTATGCAGCCAGATC-3’) and OJH1233-GR (5’-AAAGCTTTCTAGACACCGGTGGATCCACAGGTACAGTTCTGCTGGTCAATC-3’), and cloned into pJH45 (digested by *Eco*RI/*Bam*HI) by Gibson assembly.	This work
pJH1234	Eight tandem ARE-driven SEAP and mouse insulin expression in pcDNA3.1 vector (P_{ARE8}-SEAP-P2A-mINS-pA).<br>The fragment was PCR-amplified from pJH1040 with OJH1219-GF (5’-GGTACTGTTGGTAAACTCGAGGAATTCACCATGACTAGTCTGCTGC-3’) and OJH1234-GR (5’-GATCCACCGGTGTCTAGAAAGCTTTGAGGCCGGCCTGCAG-3’), and cloned into pJH1227 (digested by *Eco*RI/*Hin*dIII) by Gibson assembly.	This work

pJH1235	SB100X-specific transposon containing eight tandem ARE-driven SEAP and mouse insulin expression unit and a constitutive ECFP and PuroR expression unit BB6-PuroR.<br><br>(ITR- P_{ARE8}-SEAP-P2A-mINS-pA: P_{RPBSA}-ECFP-P2A-PuroR-pA-ITR).<br><br>The fragment was digested from pJH1234 with *Mlu*I and *Hin*dIII restriction enzymes, then recovered for T4 ligase ligation into BB6-PuroR (digested by *Mlu*I/*Hin*dIII).	This work
pJH1236	SB100X-specific transposon containing eight tandem ARE-driven SEAP and mouse insulin expression unit and a constitutive BlastR and iRFP expression unit BB6-BlastR.<br><br>(ITR- P_{ARE8}-SEAP-P2A-mINS-pA:P_{hCMV}-BlastR-P2A-iRFP-pA-ITR).<br><br>The fragment was digested from pJH1234 with *Mlu*I and *Hin*dIII restriction enzymes, then recovered for T4 ligase ligation into BB6-BlastR (digested by *Mlu*I/*Hin*dIII).	This work
pJH1237	SB100X-specific transposon containing eight tandem ARE-driven SEAP and mouse insulin expression unit and a constitutive ZeoR and mRuby expression unit BB6-ZeoR.<br><br>(ITR- P_{ARE8}-SEAP-P2A-mINS-pA: P_{hCMV}-ZeoR-P2A-mRuby-pA-ITR).<br>The fragment was digested from pJH1234 with *Mlu*I and *Hin*dIII restriction enzymes, then recovered for T4 ligase ligation into BB6-ZeoR (digested by *Mlu*I/*Hin*dIII).	This work

Abbreviations: **ARE**: antioxidant response element; **BlastR**, gene conferring blasticidin resistance; **CMV**, cytomegalovirus; **CRE**, cAMP-response element; **CREB1**, CAMP-responsive element binding protein 1; **dCas9**, nuclease-deactivated Cas9 endonuclease; **ECFP**, enhanced cyan fluorescent protein; **eGFP**, enhanced green fluorescent protein; **Elk1**, ETS like-1 transcription factor; **FLAG**, FLAG octapeptide tag; **GLP-1**, glucagon-like peptide 1; **hIR**, human insulin receptor; **iRFP**, near-infrared fluorescent protein; **ITR**, inverted terminal repeats

of SB100X; **KEAP1**: Kelch-like ECH-associated protein 1; **MCS**, multiple cloning site; **mINS**, modified insulin variant for optimal expression in HEK-293 cells; **mRuby**: a bright monomeric red fluorescent protein; **NanoLuc**, *Oplophorus gracilirostris* luciferase; **NRF2**: nuclear factor erythroid 2 p45-related factor 2; **O_{tetR}**, TetR-specific operator; **P2A**, picornavirus-derived ribosome skipping sequence optimized for bicistronic expression in mammalian cells; **pA**, polyadenylation signal; **PCR**, polymerase chain reaction; **P_{CRE}**, CRE-containing synthetic mammalian promoter; **P_{DART}**, promoter of DC-actuated regulation technology containing ARE element, O_{ARE}-$P_{hCMVmin}$; **P_{DART2}**, O_{ARE2}-$P_{hCMVmin}$; **P_{DART3}**, O_{ARE3}-$P_{hCMVmin}$; **P_{DART4}**, O_{ARE4}-$P_{hCMVmin}$; **$P_{EF-1\alpha}$**, Human elongation factor-1 alpha promoter; **P_{hCMV}**, human cytomegalovirus immediate early promoter; **$P_{hCMVmin}$**, minimal version of P_{hCMV}; **P_{mPGK}**, murine phosphoglycerate kinase promoter; **P_{RPBSA}**: a constitutive synthetic mammalian promoter; **P_{SV40}**, simian virus 40 promoter; **P_{TRE}**, O_{TetR}-$P_{hCMVmin}$; **PuroR**, gene conferring puromycin resistance; **SB100X**, optimized Sleeping Beauty transposase; **SEAP**, human placental secreted alkaline phosphatase; **shGLP1**, short human glucagon-like peptide 1; **slGFP**, short-lived eGFP; **TetR**, *Escherichia coli* Tn10-derived tetracycline-dependent repressor of the tetracycline resistance gene; **VP64**, a transcriptional activator composed of four tandem copies of VP16 (herpes simplex viral protein 16); VPR, artificial transactivation domain containing VP64-p65-Rta; **XL**, *Xenopus laevis*; **ZeoR**, gene conferring zeocin resistance.

Supplementary Table 2. qPCR primers used in this study.

Genes	Primers (Forward)	Primers (Reverse)
GAPDH (house-keeping)	5'-GTCTCCTCTGACTTCAACAGCG-3'	5'-ACCACCCTGTTGCTGTAGCCAA-3'
NRF2	5'-TGAGCCCAGTATCAGCAACA-3'	5'-CTGTGCTTTCAGGGTGGTTT-3'
KEAP1	5'-TTGGCATCATGAACGAGCTG-3'	5'-TGAAGACAGGGCTGGATGAG-3'

References:

1. Keeley, M. B.; Busch, J.; Singh, R.; Abel, T., *BioTechniques* **2005,** *39* (4), 529-536.
2. Mátés, L.; Chuah, M. K.; Belay, E.; Jerchow, B.; Manoj, N.; Acosta-Sanchez, A.; Grzela, D. P.; Schmitt, A.; Becker, K.; Matrai, J., *Nature genetics* **2009,** *41* (6), 753-761.
3. Kowarz, E.; Löscher, D.; Marschalek, R., *Biotechnology journal* **2015,** *10* (4), 647-653.
4. Kemmer, C.; Gitzinger, M.; Daoud-El Baba, M.; Djonov, V.; Stelling, J.; Fussenegger, M., *Nature biotechnology* **2010,** *28* (4), 355-360.

5. Ye, H.; Xie, M.; Xue, S.; Hamri, G. C.-E.; Yin, J.; Zulewski, H.; Fussenegger, M., *Nature biomedical engineering* **2016,** *1* (1), 0005.
6. Wyler, E.; Franke, V.; Menegatti, J.; Kocks, C.; Boltengagen, A.; Praktiknjo, S.; Walch-Rückheim, B.; Bosse, J.; Rajewsky, N.; Grässer, F., *Nature communications* **2019,** *10* (1), 4878.
7. Chavez, A.; Scheiman, J.; Vora, S.; Pruitt, B. W.; Tuttle, M.; Iyer, E. P.; Lin, S.; Kiani, S.; Guzman, C. D.; Wiegand, D. J., *Nature methods* **2015,** *12* (4), 326-328.
8. Fan, W.; Tang, Z.; Chen, D.; Moughon, D.; Ding, X.; Chen, S.; Zhu, M.; Zhong, Q., *Autophagy* **2010,** *6* (5), 614-621.
9. Mansouri, M.; Hussherr, M.-D.; Strittmatter, T.; Buchmann, P.; Xue, S.; Camenisch, G.; Fussenegger, M., *Nature communications* **2021,** *12* (1), 1-10.
10. Fussenegger, M.; Bailey, J. E.; Varner, J., *Nature biotechnology* **2000,** *18* (7), 768-774.
11. Xie, M.; Ye, H.; Wang, H.; Charpin-El Hamri, G.; Lormeau, C.; Saxena, P.; Stelling, J.; Fussenegger, M., *Science* **2016,** *354* (6317), 1296-1301.
12. Camp, N. D.; James, R. G.; Dawson, D. W.; Yan, F.; Davison, J. M.; Houck, S. A.; Tang, X.; Zheng, N.; Major, M. B.; Moon, R. T., *Journal of Biological Chemistry* **2012,** *287* (9), 6539-6550.
13. Xue, S.; Yin, J.; Shao, J.; Yu, Y.; Yang, L.; Wang, Y.; Xie, M.; Fussenegger, M.; Ye, H., *Molecular Therapy* **2017,** *25* (2), 443-455.
14. Gibson, D. G.; Young, L.; Chuang, R.-Y.; Venter, J. C.; Hutchison, C. A.; Smith, H. O., *Nature methods* **2009,** *6* (5), 343-345.
